# A novel MALDI-TOF MS-based method for blood meal identification in insect vectors: A proof of concept study on phlebotomine sand flies

**DOI:** 10.1371/journal.pntd.0007669

**Published:** 2019-09-09

**Authors:** Kristyna Hlavackova, Vit Dvorak, Alexandra Chaskopoulou, Petr Volf, Petr Halada

**Affiliations:** 1 Department of Parasitology, Faculty of Science, Charles University, Prague, Czech Republic; 2 USDA–ARS, European Biological Control Laboratory, Thessaloniki, Greece; 3 Institute of Microbiology of the Czech Academy of Sciences, Prague, Czech Republic; Instituto Oswaldo Cruz, BRAZIL

## Abstract

**Background:**

Identification of blood sources of hematophagous arthropods is crucial for understanding the transmission cycles of vector-borne diseases. Many different approaches towards host determination were proposed, including precipitin test, ELISA, DNA- and mass spectrometry-based methods; yet all face certain complications and limitations, mostly related to blood degradation. This study presents a novel method for blood meal identification, peptide mass mapping (PMM) analysis of host-specific hemoglobin peptides using MALDI-TOF mass spectrometry.

**Methodology/Principal findings:**

To identify blood meal source, proteins from abdomens of engorged sand fly females were extracted, cleaved by trypsin and peptide fragments of host hemoglobin were sequenced using MALDI-TOF MS. The method provided correct host identification of 100% experimentally fed sand flies until 36h post blood meal (PBM) and for 80% samples even 48h PBM. In females fed on two hosts, both blood meal sources were correctly assigned for 60% of specimens until 36h PBM. In a validation study on field-collected females, the method yielded unambiguous host determination for 96% of specimens. The suitability of PMM-based MALDI-TOF MS was proven experimentally also on lab-reared *Culex* mosquitoes.

**Conclusions/Significance:**

PMM-based MALDI-TOF MS analysis targeting host specific hemoglobin peptides represents a sensitive and cost-effective method with a fast and simple preparation protocol. As demonstrated here on phlebotomine sand flies and mosquitoes, it allows reliable and rapid blood source determination even 48h PBM with minimal material input and provides more robust and specific results than other currently used methods. This approach was also successfully tested on field-caught engorged females and proved to be a promising useful tool for large-scale screening of host preferences studies. Unlike other methods including MALDI-TOF protein profiling, it allows correct identification of mixed blood meals as was demonstrated on both experimentally fed and field-collected sand flies.

## Author summary

Leishmaniases belong among the most important and yet neglected vector-borne diseases, transmitted mostly by bite of female phlebotomine sand flies. To understand role of different reservoir hosts in the transmission cycles, it is important to determine blood meal sources of bloodfeeding females. Most of currently used methods face challenges due to tiny volumes of engorged blood, in case of mammals also enucleated, as well as quick progress of blood digestion which leads to rapid DNA and protein degradation. New approach towards blood source determination presented in this study is based on MALDI-TOF mass spectrometry that identifies unique peptide sequences of host hemoglobins, showing high precision and sensitivity together with a longer time period for successful host determination when compared to nowadays standardly used DNA sequencing. It was tested and verified on engorged phlebotomine sand flies from both laboratory colonies and natural endemic areas and also on *Culex* mosquitoes and shall be universal to hematophagous insects. Beside blood meal identification, it allows also the use of both morphological and molecular methods (DNA- or protein-based) for the species identification of the analysed specimen.

## Introduction

Phlebotomine sand flies (Diptera, Phlebotominae) are tiny nocturnal dipterans of high medical and veterinary importance as they are involved in transmission of several arboviruses, bacteria and protozoans that affect human and animal health. Among these, parasitic protozoans of the genus *Leishmania* are of paramount importance as they are causative agents of leishmaniases, a complex of neglected diseases with clinical manifestation ranging from cutaneous to mucocutaneous and visceral forms [1] that annually affect around 13 million people [2]. For better understanding of transmission cycles between vectors and their hosts, it is necessary to precisely identify their blood meal sources as the knowledge of reservoir animals, possible hosts and transmission dynamics of diseases is important for setting properly targeted control measures.

Phlebotomine females mostly require at least one blood meal to finish a gonotrophic cycle and lay eggs [3]. Moreover, during their life span, females can produce several egg clutches, which increases their need for blood meal and thus the possibility of disease transmission [4]. Females suck on average very small volumes of blood (up to 1.0 μl) depending on the species [5,6]. After feeding, blood is enclosed in a peritrophic matrix in mesenteron, digested by hydrolytic enzymes, mostly trypsin- and chymotrypsin-like endoproteases, and the remnants are later defecated. It was demonstrated that the speed of blood digestion significantly varies among different species, the digestion times are affected by many factors such as temperature and blood meal volumes [7,8].

Tiny volumes of blood meals together with variations in blood digestion and defecation pace pose complications to successful host identification. First methods used for blood source determination were serological techniques like precipitin test [9,10] or ELISA [11,12], which is still used nowadays. Later, DNA-based molecular methods were developed and are widely applied since; for phlebotomine sand flies the most often used is sequencing of cytochrome b (cyt b) gene [13,14], although this approach is laborious and quite expensive. Therefore, several other molecular techniques were introduced in order to simplify and optimize the workflow, but most of them were still DNA-based approaches such as multiplex PCR, PCR-RFLP, and qPCR [15]. As an alternative to DNA techniques, two approaches utilizing liquid chromatography-mass spectrometry (LC-MS/MS) were designed. The combination of non-targeted shotgun proteomics and mass spectral libraries enabled the detection of blood remnants of vertebrate hosts in ticks even six months after feeding [16]. Later, blood meals were identified by LC-MS/MS in triatomine bugs in a first application of this method on field-collected arthropod disease vectors [17]. However, none of these proteomics-based techniques became routinely applied to other hematophagous insects, probably because being time-consuming and technically demanding.

MALDI-TOF MS protein profiling represents nowadays an established method for species determination in many groups of organisms. Beside others, it was successfully applied on many hematophageous insects that serve as vectors of important infectious diseases [18] including sand flies [19]. In addition, this method was recently shown as a promising tool for host blood determination of both laboratory-reared [20] and field-collected mosquitoes [21]. Nevertheless, the approach provided successful host identification only for freshly engorged females in a short time window of 1–24 h PBM. It appears that the analysis of protein profiles of engorged mosquitoes that dynamically change as the blood digestion proceeds cannot successfully overcome a typical drawback of many other blood meal identification methods: relatively short time after the blood meal intake when conclusive identification is possible.

In the present study we applied MALDI-TOF protein profiling to determine blood meals of experimentally engorged sand flies and more importantly, we introduced a novel approach for blood meal determination, which employs peptide mass mapping (PMM)-based MALDI-TOF MS analysis [22] of host blood from abdomen digested by exogenously added trypsin. Obtained peptides, typically fragments of host hemoglobins, were detected by PMM and then sequenced on MALDI-TOF mass spectrometer, resulting in correct and reliable identification of blood origin. The efficiency and specificity of the method was first tested on sand fly females experimentally engorged on rabbit and three rodent species and its practical usefulness was illustrated in a blind study using field-collected sand fly females. Moreover, the applicability of this method on other insects was demonstrated on *Culex* mosquitoes. PMM-based MALDI-TOF mass spectrometry of trypsin-digested host blood was proven to be an accurate and robust approach for blood meal identification, especially useful for high numbers of samples collected during field surveys. The method is cost-effective and fast, requiring minimal material input and simple sample preparation, and unlike MALDI-TOF MS protein profiling it allows conclusive blood meal identification later after the blood meal intake. Although here tested on phlebotomine sand flies, the application of this method may be extended to other blood-sucking insects. Moreover, the utilization of only dissected abdomen enables other complementary methods to use remaining body parts for species identification, including DNA-based methods and vouchering of mounted head and genitalia for purposes of later morphological reference.

## Materials and methods

### Ethics statement

Animals used in this study were maintained and handled in the animal facility of Charles University, Prague in accordance with institutional guidelines and Czech legislation (Act No. 246/1992 and 359/2012 coll. on Protection of Animals against Cruelty in present statutes at large), which complies with all relevant EU guidelines for experimental animals. All experiments were approved by the Committee on the Ethics of Laboratory Experiments of the Charles University and were performed under the Certificate of Competency (Registration Number: CZ 02432).

### Insect colonies, sample storing and experimental blood feeding

All experiments were performed using sand flies from laboratory colonies reared under standardized conditions [23] in the insectary of the Department of Parasitology, Faculty of Science, Charles University. Specimens of four species were used (country of origin of females used to establish the colony is given in brackets): *P*. (*Euphlebotomus*) *argentipes* (India), *P*. (*Larroussius*) *orientalis* (Ethiopia), *P*. (*Larroussius*) *perniciosus* (Spain), and *P*. (*Phlebotomus*) *papatasi* (Turkey). Females were experimentally bloodfed on different animals from our husbandry, which were anaesthetized by ketamine/xylazine during blood feeding. All specimens were stored in 70% high quality ethanol (Merck KGaA) at -20°C.

### Sample preparation for MALDI-TOF MS protein profiling

Females of *P*. *perniciosus* were bloodfed on five different hosts: European rabbit (*Oryctolagus cuniculus*), Syrian hamster (*Mesocricetus auratus*), multimammate rat (*Mastomys natalensis*), Neumann’s grass rat (*Arvicanthis neumanni*) and SKH1 mouse (*Mus musculus*). Engorged females were kept under standard conditions and collected at various time points: 1, 12, 24, 36, 48, 60 h post-blood meal (PBM). All specimens (three samples for each collection time point and every host) were air-dried and dissected; abdomens were homogenized in 50 μl of 25% formic acid (Merck KGaA) and grounded in 1.5 ml Eppendorf tube for 15 s using a manual BioVortexer homogenizer (BioSpec) with sterile disposable pestles. The obtained homogenate was shortly centrifuged at 10 000 *g* for 15 s and 2 μl of the supernatant were mixed in a new microtube with 2 μl of MALDI matrix. One μl of the mixture was spotted on a steel target plate in duplicate and air-dried prior to MALDI-TOF MS analysis. The MALDI matrix was prepared fresh as an aqueous 60% acetonitrile/0.3% TFA solution of sinapinic acid (30 mg/ml) (Sigma-Aldrich).

For whole blood measurements, blood of all tested animals was stored with heparin at -20°C. Two μl of blood/heparin solution were resuspended in 100 μl of 25% formic acid (Merck KGaA), shortly centrifuged and mixed with the MALDI matrix as described above.

### Sample preparation for PMM-based MALDI-TOF mass spectrometry

Females of *P*. *perniciosus* were fed on rabbit, Syrian hamster, Neumann’s grass rat and SKH1 mouse, females of *P*. *orientalis* on rabbit only. Engorged females of both species were kept under standard conditions and collected 12, 24, 36, 48 and 54 h PBM (six specimens per host and time point).

Prior to PMM analysis specimens were air-dried, dissected and separated abdomens were homogenized in 1.5 ml Eppendorf tubes for 15 s in 50 μl of distilled water (Merck KGaA) using a manual BioVortexer homogenizer (BioSpec) with sterile disposable pestles. After 15 s centrifugation of the homogenate at 10 000 *g*, 10 μl of the supernatant was transferred to a new microtube containing 10 μl of 50 mM N-ethylmorpholine acetate buffer (pH 8.1; Sigma-Aldrich) and 100 ng of sequencing grade trypsin (Promega). The obtained mixture was incubated at 37°C for 20 minutes. After the digestion, 0.5 μl of the mixture was deposited on a MALDI plate in duplicate, air-dried and overlaid with 0.5 μl of MALDI matrix (aqueous 50% ACN/0.1% TFA solution of α-cyano-4-hydroxycinnamic acid; 5 mg/ml; Sigma-Aldrich).

### Sample preparation of females fed on two hosts

Females of *P*. *perniciosus* were first bloodfed on SKH1 mice, but after they started to take the blood meal, the feeding was interrupted and partially fed females were immediately moved to a second cage to continue the feeding on rabbit. Only females visibly bloodfed on mouse were transferred to feed on the rabbit for second feeding. The females which probed the rabbit and were therefore expected to feed on both hosts were kept under standard conditions and collected 12, 24, 36, 48 and 54 h PBM. One part of these females (10 specimens for each time point) was subjected to host identification using MALDI-TOF MS protein profiling and the rest was analysed by PMM-based approach (5 specimens for each time point). The samples for both types of mass spectrometric analysis were prepared according to protocols described above.

### Parallel blood meal identification by PMM-based MALDI-TOF mass spectrometry and by cytochrome b gene sequencing

Females of *P*. *argentipes* and *P*. *papatasi* were bloodfed on BALB/c mice, and *P*. *orientalis* on rabbit; all engorged females were kept under standard conditions and collected 24, 48, 60, 72, 84 and 96h PBM. Three specimens of each sand fly species per time point were analysed in parallel by PMM-based method and DNA sequencing. The samples were first prepared according to the protocol for PMM and the residual 40 μl of the homogenates were then used for DNA isolation. DNA was isolated using QIAamp DNA Mini Kit (Qiagen) following the protocol for Blood and Body Fluid Spin Protocol. Isolated DNA was amplified by PCR using the modified vertebrate-universal specific primers targeting a segment of mitochondrial DNA cyt b gene, cyt_bb1 (5’-CCA TCC AAC ATY TCA DCA TGA TGA AA-3’) cyt_bb2 (5’-GCH CCT CAG AAT GAT ATT TGK CCT CA-3’) [24]. PCR amplification was performed in total volume of 25 μl and amplified products were visualised on 1% agarose gel. The obtained products were purified by QIAquick PCR Purification Kit (Qiagen) and directly sequenced in both directions using the primers used for DNA amplification (ABI Prism BigDye Terminator Cycle Sequencing Ready Reaction Kit). For identification of the vertebrate host species, sequences were blasted against the GenBank database using BLASTn program.

### MALDI-TOF MS protein profiling and reference database creation for host blood identification

Protein mass spectra were measured on an Ultraflex III MALDI-TOF spectrometer (Bruker Daltonics) within a mass range of 3–25 kDa and with external calibration using the Bruker Protein Calibration Standard I. Each spectrum represented an accumulation of 1000 laser shots (20×50 laser shots from different positions of the sample spot). The generated spectra were visualized and compared with FlexAnalysis 3.3 software. MALDI Biotyper 3.1 software was further employed for data processing and for database creation of host blood protein profiles. At least three spectra for each host were used for the creation of reference MSP spectra with the following parameters: a maximum peak number of 100, S/N ratio above 3, and a minimum intensity of 1% of the most intense peak. The desired peak frequency for MSP reference spectra was 70%.

### Identification of blood meal sources by PMM-based MALDI-TOF mass spectrometry

Peptide mass mapping spectra were acquired on an Ultraflex III MALDI-TOF instrument in the mass range of 700–4000 Da and calibrated externally using a peptide standard PepMix II (Bruker Daltonics). At least two peptides per sample were selected for MS/MS sequencing using LIFT technology. MS/MS data were searched against the SwissProt20171124 database subset of vertebrate proteins using in-house MASCOT search engine (Matrix Science) with the following search settings; enzyme: trypsin, variable modification: oxidation (M), MS mass tolerance: 20 ppm, MS/MS mass tolerance: 0.6 Da, number of missed cleavages: 1. MS/MS spectra with a MOWSE score over the threshold of 25 (calculated for the settings used) were considered as reliably identified. Peptide sequences of Neumann’s grass rat hemoglobin were derived by de novo MS/MS sequencing. One MALDI peptide mass map (sample EME8) was also measured on a Solarix FT-ICR mass spectrometer (Bruker Daltonics) with mass accuracy below 1 ppm.

### Blind study using field-collected sand fly females

The accuracy and usefulness of blood source identification by PMM-based approach was tested in a blind study carried on field-caught engorged females collected during a field survey in Greece (July 2017). Sand flies were sampled using carbon-dioxide (dry ice) baited CDC light traps (John W. Hock) operated overnight (approx. 5 p.m.-7 a.m.) and placed inside or next to animal shelters. Engorged females were separated and examined under a stereomicroscope; in total, 54 individuals from Greece were included in the assay. All specimens were kept in 70% pure ethanol (Merck KGaA) after the collections and stored at-20°C for several weeks prior to further analysis.

Females were processed as followed: head and genitalia were slide-mounted in CMCP-9 mounting medium (Polyscience) and morphological identification was carried out using published morphological keys [25]. The species determination was confirmed by MALDI-TOF MS protein profiling [19] using our in-house database currently comprising reference spectra of 23 different sand fly species. Abdomens were subjected to host blood determination by PMM-based method and using cyt b gene sequencing according to protocols described above.

### Host blood identification in *Culex* mosquitoes by PMM-based MALDI-TOF mass spectrometry

Laboratory-reared females of *Culex* (*Culex*) *quinquefasciatus* were bloodfed on BALB/c mice. The engorged females were collected at 12, 24, 36, 48, 60 h PBM and stored in 70% high quality ethanol (Merck KGaA) at -20°C (five females for each time point). The specimens were air-dried, dissected abdomens were homogenized in 200 μl of water and further prepared for PMM analysis according to the protocol described above for sand flies.

## Results

### Host blood identification by MALDI-TOF MS protein profiling

**Fig 1:**
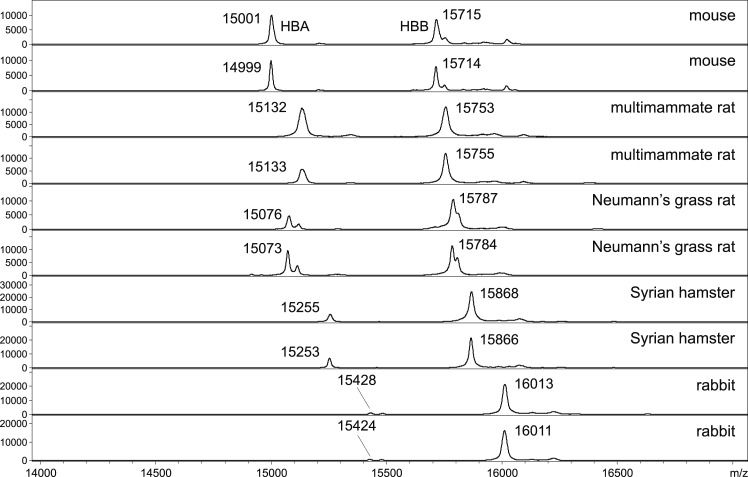
MALDI-TOF protein profiles of homogenized female abdomens of *P*. *perniciosus* bloodfed on five different hosts 12 h after feeding. Peaks of host hemoglobin subunit alpha (HBA; about 15 kDa) and hemoglobin subunit beta (HBB; about 16 kDa) dominated in the spectra. For each host the spectra of two females are presented.

To evaluate the potential of MALDI-TOF MS protein profiling for blood meal source identification in phlebotomine sand flies, protein spectra of homogenized abdomens of *P*. *perniciosus* females bloodfed on five different animals from our husbandry (SKH1 mouse, Syrian hamster, Neumann’s grass rat, multimammate rat or rabbit) were analysed. The spectra obtained from engorged abdomens collected 12 h PBM exhibited a high degree of reproducibility for each host tested and matched with whole blood protein patterns of animals used for sand fly feeding, indicating that the abdominal spectra were specific according to host blood origin. The comparison of spectra from engorged abdomens of all hosts revealed that the protein profiles were host-specific and distinguishable according to the source of blood meal (Figs 1 and S1). The obtained spectra were rather simple, with two dominating signals of alpha- (15 kDa) and beta-subunit (16 kDa) of hemoglobin [26] that is one of the most abundant proteins of vertebrate blood.

**Fig 2:**
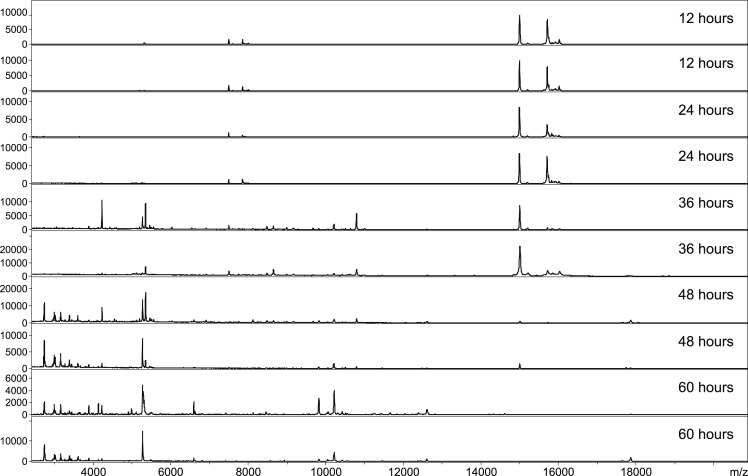
MALDI-TOF protein profiles of homogenized female abdomens of *P*. *perniciosus* bloodfed on SKH1 mouse at different time points after feeding. The fragments of hemoglobin were produced by blood digestion in the 24-36h PBM time interval. For each time point the spectra of two females are presented.

Protein profiles of abdomens from *P*. *perniciosus* females bloodfed on five experimental hosts sampled every 12 h from 1 h until 60 h PBM were subjected to MALDI-TOF MS to assess the effectivity of the method for determination of host blood origin in the course of time post blood feeding. As shown in Fig 2, the abdominal spectra of freshly engorged females were identical and stable from 1 h to 24 h PBM. Changes of protein patterns are evident at 36 h PBM and were more significant at 48 h and 60 h PBM time points with practically no detectable signals of intact hemoglobins. These alterations occurred regardless of the blood source (S2 Fig), presumably due to ongoing digestion of host blood in sand fly gut. Based on these results, a reference database was created from spectra of engorged abdomens collected 12 h PBM. Three samples for each collection time point and every host were further analysed by MALDI-TOF MS (in total 90 specimens) and the acquired protein profiles were queried against this database. The database search yielded 100% correct determination of blood meal source for all 45 specimens collected until 24 h PBM. For 36 h PBM time point, only 6 of 15 females gave accurate identification of blood meal origin. Remaining nine individuals were misidentified because of spectra altered by proceeding blood digestion. As expected, the substantially changed protein profiles made successful identification impossible for females collected 48 h and 60 h after feeding.

### Host blood identification by PMM-based MALDI-TOF mass spectrometry

As an alternative to MALDI-TOF MS protein profiling, peptide mass mapping (PMM) using MALDI-TOF MS was adapted for blood source determination in sand flies.

**Fig 3:**
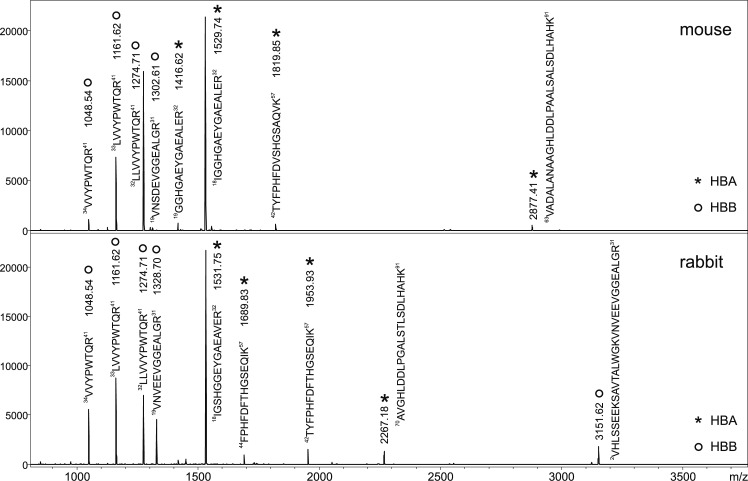
MALDI-TOF peptide mass maps measured after trypsin digestion of homogenized abdomens of *P*. *perniciosus* fed on SKH1 mouse and rabbit. The peaks are labelled by corresponding peptide sequences (see Table 1) of host HBA (asterisks) and HBB (circles). In comparison to the Table 1 a double mutation in the peptide AA 63–91 was detected for SKH1 mouse used in this study as a blood source. The bloodfed females were collected 12 h PBM.

**Fig 4:**
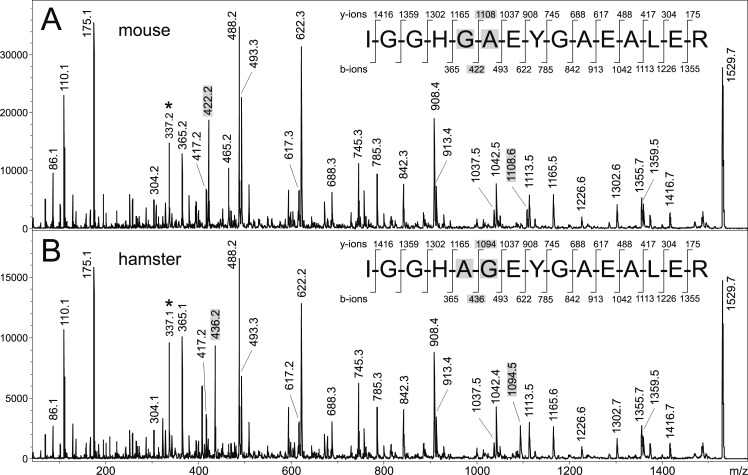
Host blood identification using MS/MS sequencing of trypsin-generated peptides from homogenized engorged abdomens. The peptide sequences of host hemoglobin were proved by almost complete series of b- and y-type fragment ions [22]. The approach is even capable to distinguish isobaric peptide sequences as shown on the example of HBA fragment from SKH1 mouse (A) and hamster (B) with the same protonated mass [M+H]^+^ = 1529.7. The peptides differ in only two amino acids which are highlighted in grey, as well as the ions verifying those differences. The a_4_ fragment ions are marked by asterisks.

Peptide mass maps of trypsin-digested abdomens of *P*. *pernicious* females fed on four different vertebrates (rabbit, Syrian hamster, Neumann’s grass rat and SKH1 mouse) were found distinct, displaying several host-specific signals of alpha- and beta-hemoglobin peptides (Figs 3 and S3). As hemoglobin sequences of potential host animals differ, characteristic peptide fragments serving as a unique host signature could be easily generated by trypsin digestion for each animal (Table 1). The sequencing of the fragments by tandem mass spectrometry (MS/MS) and subsequent database searching allowed to determine amino acid sequence of these discriminating peptides and thus to provide the correct assignment of blood meal origin. Interestingly, MS/MS sequencing was also able to distinguish isobaric peptides (those having the same mass) originating from different hosts (Fig 4).

**Table 1:** Unique tryptic peptides of host hemoglobins used for blood meal source identification using PMM-based MALDI-TOF mass spectrometry.

**Hemoglobin subunit alpha**					
**Host**	**DTB No.**	**AA 18–32**	**[M+H]**^**+**^	**AA 63–91 / 70–91**	**[M+H]**^**+**^
Human	P69905	VGAHAGEYGAEALER	1529.73	VADALTNAVAHVDDMPNALSALSDLHAHK	2996.49
Mouse	P01942	IGGHGAEYGAEALER	1529.73	VADALASAAGHLDDLPGALSALSDLHAHK	2836.46
Rabbit	P01948	IGSHGGEYGAEAVER	1531.71	AVGHLDDLPGALSTLSDLHAHK	2267.18
Hamster	P01945	IGGHAGEYGAEALER	1529.73	VADALTNAVGHLDDLPGALSALSDLHAHK	2921.51
Grass rat	NA	IGGHGGEYGAEALER	1515.72	VADALANAASHLDDLPGALSALSDLHAHK	2893.48
Cow	P01966	VGGHAAEYGAEALER	1529.73	AVEHLDDLPGALSELSDLHAHK	2367.19
Sheep	P68240	VGGNAGAYGAEALER	1434.70	AVGHLDDLPGTLSDLSDLHAHK	2311.17
Goat	P0CH25	VGGNAGAYGAEALER	1434.70	AVGHLDDLPGTLSDLSDLHAHK	2311.17
Dog	P60529	IGGHAGDYGGEALDR	1487.69	VADALTTAVAHLDDLPGALSALSDLHAYK	2948.54
Cat	P07405	IGSHAGEYGAEALER	1559.75	VADALTQAVAHMDDLPTAMSALSDLHAYK	3055.49
Chicken	P01994	IAGHAEEYGAETLER	1645.78	VVAALIEAANHIDDIAGTLSKLSDLHAHK	3022.63
**Hemoglobin subunit beta**					
**Host**	**DTB No.**	**AA 17–29 / 19–31**	**[M+H]**^**+**^	**AA 40–58 / 42–60 / 42–62**	**[M+H]**^**+**^
Human	P68871	VNVDEVGGEALGR	1314.66	FFESFGDLSTPDAVMGNPK	2058.95
Mouse	P02088	VNSDEVGGEALGR	1302.63	YFDSFGDLSSASAIMGNAK	1980.90
Rabbit	P02057	VNVEEVGGEALGR	1328.68	FFESFGDLSSANAVMNNPK	2074.95
Hamster	P02094	VNADAVGAEALGR	1242.64	FFEHFGDLSSASAVMNNPQVK	2325.10
Grass rat	NA	VNADQVGGEALGR	1285.65	YFDNFGDLSSASAIMGNAK	2007.91
Cow	P02070	VKVDEVGGEALGR	1328.71	FFESFGDLSTADAVMNNPK	2089.95
Sheep	P02075	VKVDEVGAEALGR	1342.73	FFEHFGDLSNADAVMNNPK	2152.98
Goat	P02077	VKVDEVGAEALGR	1342.73	FFEHFGDLSSADAVMNNAK	2099.95
Dog	P60524	VNVDEVGGEALGR	1314.66	FFDSFGDLSTPDAVMSNAK	2048.93
Cat	P07412	VNVDEVGGEALGR	1314.66	FFESFGDLSSADAIMSNAK	2036.93
Chicken	P02112	VNVAECGAEALAR	1302.65	FFASFGNLSSPTAILGNPMVR	2226.14

The sequences and masses of hemoglobin peptides were generated using a PeptideMass tool [27]. Two peptide sequences from each hemoglobin subunit are shown for every host. The hemoglobin peptides for grass rat were identified by de novo MS/MS sequencing.

DTB No., UniProtKB database number; AA; amino acid. [M+H]^+^; mass of protonated peptide ion; NA; not available.

**Fig 5:**
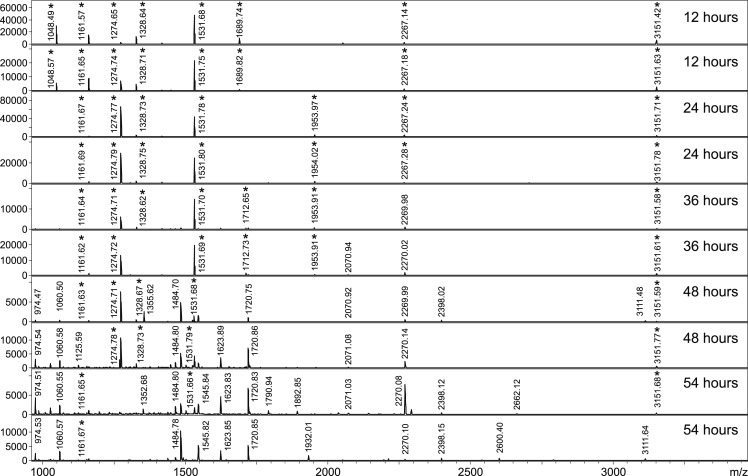
Abdominal peptide mass spectra of *P*. *orientalis* females at different time points after blood feeding on rabbit. The peptide signals of rabbit hemoglobin are marked by asterisks. The peaks related to digestion of sand fly body become obvious since 48h PBM. For each time point the spectra of two females are presented.

To evaluate the usefulness of PMM-based methodology for assignment of host blood origin, females of *P*. *perniciosus* fed either on rabbit, hamster, Neumann’s grass rat or SKH1 mouse and *P*. *orientalis* engorged on rabbit were tested. The specimens of both sand fly species were sampled at 12, 24, 36, 48, and 54 h after feeding. The spectra of all females collected until 24 h PBM consisted exclusively of peptides of host hemoglobin as shown on tryptic maps of *P*. *perniciosus* fed on rabbit and mouse (Fig 3) and also demonstrated for *P*. *orientalis* engorged on rabbit (Fig 5). At 36 h PBM, minor changes were observed and tiny signals of the peptide fragments originating from sand fly body were detected. These peaks became more visible 48 h PBM and were quite dominant in the spectra of females collected 54 h after feeding regardless of the blood source and vector species (Figs 5 and S4). Nevertheless, the presence of arthropod-related signals did not prevent successful determination of blood meal origin because host identification using PMM-based approach, in contrary to MS protein profiling, was not dependent on overall spectrum pattern.

In the proof of concept study focused on determination of blood meal source in large group of sand fly females, six specimens per condition depicted above were subjected to PMM-based MALDI-TOF MS, namely 30 specimens of *P*. *orientalis* engorged on rabbit and sampled at five different points in time after feeding, and 120 females of *P*. *perniciosus* bloodfed on four animals (30 samples per host). Using PMM method, the origin of host blood was properly identified for all 90 specimens (100%) up to 36 h PBM. At 48 h PBM, 24 of 30 females (80%) yielded unambiguous assignment of blood meal source and finally 50% correct identification (15/30) was achieved for females collected 54 h after engorgement.

### Identification of blood sources in females fed on two hosts

Females of *P*. *perniciosus* were bloodfed consecutively on two host animals (first on mouse, then on rabbit) and collected 12, 24, 36, 48, and 54 h PBM. One part of the samples was subjected to host identification using MALDI-TOF MS protein profiling, the second one was analysed by PMM-based MALDI-TOF mass spectrometry.

MALDI-TOF MS protein profiling failed to determine both hosts used for the feeding in all cases, probably because of the spectra complexity. As shown on S5 Fig, the spectra of females engorged on two animals consisted of hemoglobin peaks of both hosts and represented a superposition of individual host protein profiles. However, when searching such spectra against the reference database, only identification of one host was achieved. One host species was determined for all 10 females collected 12 h PBM (mouse in nine cases, once rabbit), whereas at the time point of 24 h PBM, only 3 of 10 females were assigned with a mouse as blood source (S1 Table). At later time points, significant alterations of protein profiles related to blood degradation were observed and no hosts were determined since 36 h PBM.

Compared to MALDI-TOF MS protein profiling, PMM-based approach provided more promising results in terms of detection of multiple blood sources in sand fly females (S1 Table). The method successfully revealed both hosts in 9 of 15 (60%) females until 36 h PBM. For the remaining 6 samples, only a single host was identified (five times mouse, once rabbit). Just one blood source was determined for 5 females collected 48 h after feeding. Four of them were assigned with a rabbit host even though they definitely fed on mice as well as they were optically observed as partially engorged after first exposure to a mouse host. This finding might be ascribed to a small volume of mouse blood engorged or eventually to a different digestion rate of mouse and rabbit blood. Finally, the spectra of the specimens sampled 54 h PBM did not contain any signals of hemoglobin peptides and therefore no hosts were identified.

### Parallel identification of host blood by PMM-based MALDI-TOF mass spectrometry and using cytochrome b sequencing

The effectiveness of PMM-based MALDI-TOF mass spectrometry for identification of blood meal origin was compared with DNA sequencing, which nowadays represents the conventional method for host determination in arthropod vectors. In the comparative experiment, females of *P*. *argentipes* and *P*. *papatasi* bloodfed on BALB/c mouse, and *P*. *orientalis* engorged on rabbit were analysed in parallel by PMM approach and DNA sequencing; the species included into this assay were selected upon their known different pace of blood digestion, that was demonstrated to be fastest for *P*. *argentipes* and slowest for *P*. *orientalis* [6].

Both tested methods successfully assigned hosts of all females collected 24 h PBM, but remarkable differences were observed between these methods at 48 h PBM (Table 2). For this time point, PMM correctly determined blood meal sources of all samples of *P*. *papatasi* and *P*. *orientalis*, however in case of *P*. *argentipes*, just one of three specimens gave an identifiable spectrum due to advanced blood degradation. On the other hand, sequencing of cyt b gene was even more affected by blood digestion and yielded correct host identification only for single female of each sand fly species. For specimens sampled 60 h after feeding, no hosts were identified by DNA sequencing, whereas 3 of 9 females (two *P*. *orientalis*, one *P*. *papatasi*) were assigned with blood meal sources using PMM method. At later time points (72, 84 and 96 h PBM) no host identifications were possible, either using cyt b sequencing or by PMM approach.

**Table 2:** Parallel host identification using PMM-based MALDI-TOF mass spectrometry and cyt b sequencing.

Sand fly species	Host	PBM time	No. PMM host ID (%)	No. Cyt b host ID (%)
*P*. *argentipes*	mouse	24 h	3 (100%)	3 (100%)
		48 h	1 (33%)	1 (33%)
		60 h	0	0
*P*. *orientalis*	rabbit	24 h	3 (100%)	3 (100%)
		48 h	3 (100%)	1 (33%)
		60 h	2 (66%)	0
*P*. *papatasi*	mouse	24 h	3 (100%)	3 (100%)
		48 h	3 (100%)	1 (33%)
		60 h	1 (33%)	0

Females of *P*. *argentipes* and *P*. *papatasi* fed on BALB/c mouse, and *P*. *orientalis* on rabbit were collected at various time points after feeding (three specimens per condition) and subjected to PMM-based MALDI-TOF MS and cyt b sequencing method. The table shows results until 60h PBM, later all identification attempts were negative.

### Blind study using field-collected sand fly females

**Fig 6:**
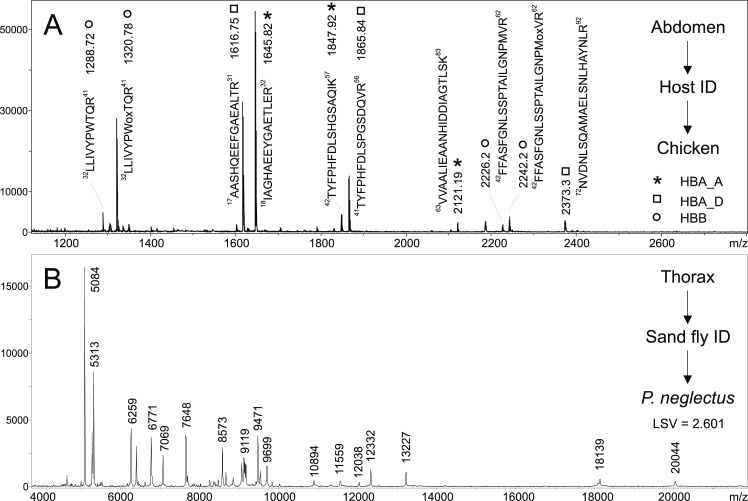
Parallel host blood and sand fly species identification from a single specimen. Blood meal source of the field-caught female EPO1 (see Table 3) was determined by PMM-based MALDI-TOF MS of trypsin-digested abdomen (A). The peaks in MALDI-TOF peptide mass map are labelled by amino acid sequences of chicken HBA (two allelic variants A and D) and HBB. Simultaneously, thorax of the specimen was utilized for sand fly species identification using MALDI-TOF protein profiling (B).

The practical usefulness of PMM-based MALDI-TOF mass spectrometric method for host determination in entomological surveys was assessed in a blind test aimed at field-collected sand flies. In total, 54 field-caught engorged females from Greece (collected in 2017) were included in the study. In addition to assignment of blood meal origin using PMM-based method, sand fly species identification was carried out by combination of morphological analysis and MALDI-TOF MS protein profiling using our database of sand fly reference spectra. Thus, both host and sand fly identifications using MALDI-TOF mass spectrometric analyses (Fig 6), by cyt b gene sequencing or morphology were performed from single sand fly specimens.

For Greek field specimens, PMM-based approach successfully determined blood meal origin in 52 out of these 54 females (Tables 3 and S2). The method failed only in two cases, probably because of low volume of engorged blood or due to advanced blood digestion. According to the results obtained using PMM method, the most common hosts were sheep (39), followed by goats (7), dogs (3), and hen (1). Furthermore, mixed blood meals were detected in two specimens as these females presumably fed on goat as well as on sheep (S6 and S7 Figs). To verify the accuracy of the results from PMM-based MALDI-TOF mass spectrometry, DNA was isolated from all 54 females and a segment of host cyt b gene was attempted to amplify. Probably because of low DNA amount, only 32 specimens yielded DNA sequence matching to a host animal in GenBank repository (Table 3). In all 32 cases, DNA sequencing confirmed the blood meal origin identified by PMM indicating a nice agreement between the results provided by both methods. In case of mixed blood meals of two females, DNA sequencing was able to determine just goat. For the remaining 22 females, cyt b sequencing identified DNA sequence related to either sand fly or to human contamination.

**Table 3:** List of field-caught engorged females from Greece.

Code	Locality	PMM host identification	Cyt b host identification	MALDI-TOF MS species identification	Morphological species identification
EAG1	Agriacona	*Capra hircus*	*Capra hircus*	*P*. *perfiliewi*	*P*. *tobbi / P*. *perfiliewi*
EAG21*	Agriacona	*Capra hircus*	*Capra hircus*	*P*. *neglectus*	*P*. *neglectus*
EAG22	Agriacona	*Capra hircus*	*Capra hircus*	*P*. *neglectus*	*P*. *neglectus*
EAG23	Agriacona	*Canis lupus familiaris*	n.d.	*P*. *perfiliewi*	*P*. *perfiliewi*
ELE6	Levidi	*Ovis aries*	*Ovis aries*	*P*. *neglectus*	*P*. *neglectus*
ELE7	Levidi	*Canis lupus familiaris*	n.d.	*P*. *tobbi*	*P*. *tobbi / P*. *perfiliewi*
ELE8	Levidi	*Ovis aries*	n.d.	*P*. *tobbi*	*P*. *tobbi / P*. *perfiliewi*
ELE9	Levidi	*Ovis aries*	n.d.	*P*. *neglectus*	*P*. *neglectus*
ELE10	Levidi	*Ovis aries*	n.d.	*P*. *neglectus*	*P*. *neglectus*
ELE11*	Levidi	*Ovis aries*	*Ovis aries*	§*P*. *neglectus*	*P*. *neglectus*
ELO1	Leontari	*Ovis aries*	*Capra hircus*	*P*. *neglectus*	*P*. *neglectus*
ELV31*	Levidi / Vlacherna	n.d.	n.d.	n.d.	*P*. *neglectus*
ELV32*	Levidi / Vlacherna	*Capra hircus*	*Capra hircus*	§*P*. *neglectus*	*P*. *neglectus*
EME1	Metamorphose	*Ovis aries*	n.d.	*P*. *tobbi*	*P*. *tobbi / P*. *perfiliewi*
EME2	Metamorphose	*Ovis aries*	*Capra hircus*	*P*. *tobbi*	*P*. *tobbi / P*. *perfiliewi*
EME3	Metamorphose	*Ovis aries*	*Ovis aries*	*P*. *tobbi*	*P*. *tobbi / P*. *perfiliewi*
EME4	Metamorphose	*Ovis aries*	*Ovis aries*	*P*. *tobbi*	*P*. *tobbi / P*. *perfiliewi*
EME5	Metamorphose	*Ovis aries*	*Ovis aries*	*P*. *tobbi*	*P*. *tobbi / P*. *perfiliewi*
EME6	Metamorphose	*Ovis aries*	*Ovis aries*	*P*. *neglectus*	*P*. *neglectus*
EME7	Metamorphose	*Ovis aries*	n.d.	*P*. *tobbi*	*P*. *tobbi / P*. *perfiliewi*
EME8	Metamorphose	*Ovis aries + Capra hircus*	*Capra hircus*	*P*. *neglectus*	*P*. *neglectus*
EME9	Metamorphose	*Ovis aries*	*Ovis aries*	*P*. *tobbi*	*P*. *tobbi / P*. *perfiliewi*
EME10	Metamorphose	*Ovis aries*	n.d.	*P*. *tobbi*	*P*. *tobbi / P*. *perfiliewi*
EME11	Metamorphose	*Ovis aries*	*Ovis aries*	*P*. *neglectus*	*P*. *neglectus*
EME12	Metamorphose	*Ovis aries*	n.d.	*P*. *tobbi*	*P*. *tobbi / P*. *perfiliewi*
EME13*	Metamorphose	*Capra hircus*	n.d.	*P*. *tobbi*	*P*. *tobbi / P*. *perfiliewi*
EME14	Metamorphose	*Ovis aries*	n.d.	*P*. *tobbi*	*P*. *tobbi / P*. *perfiliewi*
EME15	Metamorphose	*Ovis aries*	n.d.	*P*. *tobbi*	*P*. *tobbi / P*. *perfiliewi*
EME16	Metamorphose	*Ovis aries*	*Ovis aries*	*P*. *neglectus*	*P*. *neglectus*
EME17	Metamorphose	*Ovis aries*	*Ovis aries*	*P*. *tobbi*	*P*. *tobbi / P*. *perfiliewi*
EME18	Metamorphose	*Ovis aries*	n.d.	*P*. *tobbi*	*P*. *tobbi / P*. *perfiliewi*
EME19	Metamorphose	*Ovis aries*	n.d.	*P*. *tobbi*	*P*. *tobbi / P*. *perfiliewi*
EME20*	Metamorphose	*Ovis aries*	n.d.	*P*. *neglectus*	*P*. *neglectus*
EPA10	Palaio Pyrgos	*Capra hircus*	*Capra hircus*	*P*. *neglectus*	*P*. *neglectus*
EPE1*	Pelopio	*Ovis aries*	*Ovis aries*	*P*. *neglectus*	*P*. *neglectus*
EPE2	Pelopio	*Ovis aries*	n.d.	*P*. *neglectus*	*P*. *neglectus*
EPK1	Pakia	*Ovis aries*	*Ovis aries*	*P*. *tobbi*	*P*. *tobbi / P*. *perfiliewi*
EPK2	Pakia	*Ovis aries*	*Ovis aries*	*P*. *neglectus*	*P*. *neglectus*
EPK3*	Pakia	n.d.	n.d.	*P*. *tobbi*	*P*. *tobbi / P*. *perfiliewi*
EPK4	Pakia	*Ovis aries*	*Ovis aries*	*P*. *tobbi*	*P*. *perfiliewi*
EPK5	Pakia	*Canis lupus familiaris*	n.d.	*P*. *tobbi*	*P*. *perfiliewi*
EPK6	Pakia	*Ovis aries + Capra hircus*	*Capra hircus*	*P*. *neglectus*	*P*. *neglectus*
EPO1	Agriacona	*Gallus gallus*	*Gallus gallus*	*P*. *neglectus*	*P*. *neglectus*
EPO14	Agriacona	*Ovis aries*	n.d.	*P*. *tobbi*	*P*. *tobbi / P*. *perfiliewi*
EPP1	Papari	*Ovis aries*	*Ovis aries*	*P*. *neglectus*	*P*. *neglectus*
EPP2	Papari	*Ovis aries*	*Ovis aries*	*P*. *neglectus*	*P*. *neglectus*
EPP3	Papari	*Ovis aries*	n.d.	*P*. *neglectus*	*P*. *neglectus*
EPP4	Papari	*Ovis aries*	*Ovis aries*	*P*. *neglectus*	*P*. *neglectus*
EPP5*	Papari	*Ovis aries*	*Ovis aries*	§*P*. *neglectus*	*P*. *neglectus*
EPT1*	Asteri	*Ovis aries*	*Ovis aries*	*P*. *perfiliewi*	*P*. *tobbi/P*. *perfiliewi*
EPT2	Asteri	*Ovis aries*	*Ovis aries*	*P*. *tobbi*	*P*. *tobbi*
EPT20*	Asteri	*Ovis aries*	*Ovis aries*	*P*. *neglectus*	*P*. *neglectus*
EPT21	Asteri	*Ovis aries*	*Ovis aries*	*P*. *perfiliewi*	*P*. *perfiliewi*
EST1*	Stefania	*Capra hircus*	n.d.	§*P*. *papatasi*	*P*. *papatasi*

* Females with the damaged peritrophic matrix.

^§^ Specimens with uncertain species identification.

n.d.; not determined.

Besides blood meal source determination, sand fly species identification of all Greek females was successfully performed by combination of morphological analysis and MALDI-TOF MS protein profiling (Table 3). *P*. *neglectus* (26) and *P*. *tobbi* (23) were revealed as the most abundant species, other species found were *P*. *perfiliewi* (4) and *P*. *papatasi* (1). Twelve of 54 analysed specimens had damaged peritrophic matrixes and blood present in thorax negatively affected their MALDI-TOF protein profiles. Only 7 of these 12 females yielded reliable sand fly determination with log score value (LSV) higher than 2.0. Four samples with low quality spectra gave correct, but uncertain species identification with LSV below 2.0, and one specimen was not assigned at all.

### Host blood identification in *Culex* mosquitoes by PMM-based MALDI-TOF mass spectrometry

**Fig 7:**
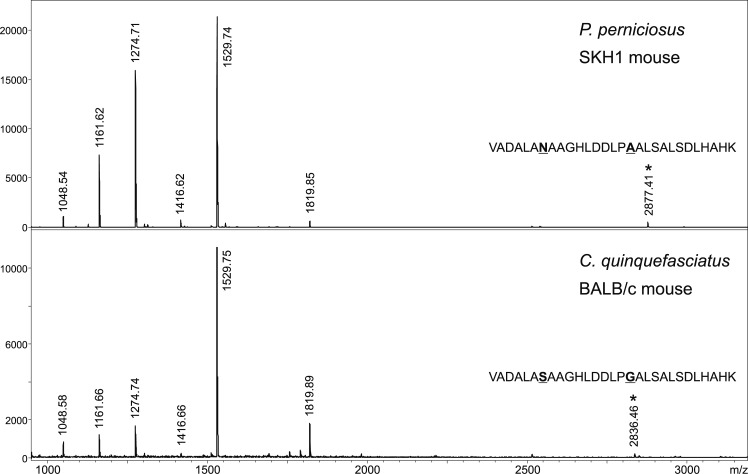
Comparison of abdominal spectra of sand fly (*P*. *perniciosus*) and mosquito (*C*. *quinquefasciatus*) females fed on mice. Practically identical MALDI-TOF peptide mass maps were observed for both insects. The only difference was caused by a double mutation within a peptide sequence of HBA (marked by asterisks), which was related to two types of mouse used as a blood source (SKH1 vs. BALB/c). The engorged females were collected 12 h PBM.

To demonstrate the universality of PMM-based MALDI-TOF mass spectrometry as a tool for host identification in blood-sucking insects, the method was also employed for determination of blood meal origin in mosquito specimens (*C*. *quinquefasciatus*). The females were bloodfed on mice and collected 12, 24, 36, 48, and 60 h PBM (5 specimens for each time point). Until 48 h after feeding, 19 of 20 engorged females were correctly assigned with a mouse host using PMM method, which was in accordance with the results obtained for sand flies. For one mosquito (48 h PBM) the determination was not possible anymore, most likely because of advanced blood degradation. At 60 h PBM time point the blood source of only one female was identified. Interestingly, abdominal peptide profiles of sand fly (*P*. *perniciosus*) and mosquito (*C*. *quinquefasciatus*) females fed on mice were found nearly identical (Fig 7), which confirmed that the spectra of trypsin-digested abdomens of freshly engorged females are characteristic for blood meal only and not affected by arthropod species.

## Discussion

To elucidate transmission cycles of vector-borne diseases and potential reservoir hosts it is necessary to choose a suitable method for blood meal identification in order to understand the feeding preferences of studied vectors. Accuracy, speed, and cost-effectiveness are the main attributes considered when selecting such method. A crucial parameter affecting the successful blood source determination is a time delay allowed after feeding which can undermine the success of the identification due to proceeding DNA and protein degradation. The second limitation stems from a considerably low volume of engorged blood, which in phlebotomine sand flies varies from 0.1 to 0.9 μl depending on a species [5,6]. Therefore, the detection of miniscule amounts of host DNA using DNA-based techniques remains quite challenging and susceptible to DNA contamination and may lead to co-amplification of both host and sand fly DNA during PCR followed by multiple sequence reading. Moreover, the low volume of enucleated mammalian blood is one of the reasons to use mitochondrial genes for DNA-based host identifications [15], which in case of accidental contamination may cause misidentification of blood meal source as human. In our work, we observed all these drawbacks characteristic for DNA techniques and the poor results of cyt b sequencing we obtained were in agreement with the previous studies struggling to identify blood meal sources of sand fly females using DNA sequencing [28–31].

An alternative approach taking advantage of protein mass spectrometry might overcome these obstacles. One of the first proposed LC-MS/MS techniques used direct spectral matching to detect remnants of blood in ticks and enabled to track the blood sources six months back after feeding [32]. Recently, successful blood meal source identification using LC-MS/MS analysis with subsequent database search was presented in the case of field-caught triatomine bugs [17]. Despite the high sensitivity, both LC-MS/MS-based methods are quite laborious and require expertized interpretation of results. On the other hand, MALDI-TOF MS protein profiling may be a promising choice due to its simple sample preparation, speed of analysis and easy data evaluation. In the last decade, it became an established method for routine species identification of different organisms including many parasitic arthropods [18] and it was also recently tested for host blood determination for the first time on *Anopheles* mosquitoes [20].

Our study proved that MALDI-TOF MS protein profiling can be applied to identification of blood meal origin also in phlebotomine sand flies. Until 24 h after feeding, the abdominal protein profiles of females experimentally engorged on five different mammalian hosts were well reproducible and distinguishable according to blood source. The spectra allowed differentiating even hosts that belong to the same family, like different rodent species belonging to Muridae (mouse, Neumann’s grass rat and multimammate rat). However, since 36 h PBM, obvious changes of protein spectra induced by blood degradation were observed which dramatically decreased the effectiveness of MALDI-TOF MS protein profiling for host blood identification similarly as reported for DNA-based techniques [33,34].

Beside the time frame within which the blood meal identification is still possible, another key parameter affecting the correctness of host identification is a specificity of the used method, which in case of MALDI-TOF protein profiling might not be sufficient to cope with intraspecific variability caused by genetic polymorphism or related to possible hemoglobin isoforms. Each single point mutation or a post-translational modification of hemoglobin sequence induces an inevitable shift of protein molecular weight that might unable determination of blood meal origin using the method relying on database search against reference protein masses. Vice versa, the host identification could also fail in those host organisms which, despite their hemoglobin sequences differing, have the same or very close total mass of hemoglobin subunits. In addition, a need for a specific database that would include protein spectra of most potential host species from a sampled geographical area is yet another limitation of the protein profiling approach.

To overcome these drawbacks, we introduced a novel method based on MALDI-TOF peptide mass mapping (PMM) mass spectrometry. The analysis is targeted on hemoglobin, one of the most abundant proteins in blood. Although highly conserved across the taxa, hemoglobin also displays small variations in the sequence that can be used for precise host blood identification at the species level [35]. The PMM method is primarily known as a tool for protein identification utilizing a proteolytic digestion of protein of interest followed by mass spectrometric analysis of resulting peptide mixture [22]. The measured masses of the detected peptides are compared to the predicted ones which can be generated from known protein sequence using a program such as PeptideMass [27]. The recorded spectrum, called peptide mass map, represents set of peptide signals characteristic not only for the protein in question, but also for the organism from which the protein originates. The sequences of these specific peptide fragments, here the peptides of host hemoglobin, are then determined by MS/MS sequencing using MALDI-TOF mass spectrometer in order to achieve the blood meal source identification. This approach, unlike MALDI-TOF MS protein profiling, does not need a creation of a specific database since it employs already existing public ones such as UniProt protein database. The proposed PMM-based MALDI-TOF MS method is very simple and fast not requiring any LC separation prior to MS like LC-MS/MS-based approaches. The sample is measured directly after trypsin digestion without any purification step and host identification of analyzed specimen is completed in less than one hour. As several individuals can be processed in parallel, the real throughput of the method is in fact much higher.

The effectiveness of PMM-based MALDI-TOF MS for blood meal source determination was first demonstrated in the proof of concept study using sand fly females fed on rabbit or various small rodents. In comparison to both MALDI-TOF MS protein profiling and DNA sequencing, the PMM-based approach was found less affected by blood degradation and allowed reliable host assignment for longer time period after engorgement. The method yielded confident blood meal identification until 48 h PBM and for some females even 54 h after feeding, which clearly outmatched the conventional PCR-based protocols [36,37]. In addition, it was revealed that the success rate of host identification for the later time points after feeding was in accordance with the speed of blood digestion of the analyzed sand fly [6]. The PMM-based technique also provided promising results in the analysis of mixed blood meals which represent a challenge in host blood identification. As reviewed [15], the successful detection of multiple blood sources depends on several factors such as engorged volume of individual blood meals, time span between feedings, and blood digestion rate, which differs among various bloods [38] and vector species [6]. The data obtained on *P*. *perniciosus* females experimentally fed consecutively on two host animals clearly showed a potential of the method for the identification of mixed blood meals as both hosts were uncovered in 60% of tested females (9 of 15) until 36h PBM. Only a single host (five times mouse, once rabbit) was determined for the remaining six specimens. The prevailing identification of a mouse host might be due to the fact that the females, after interrupting engorgement on mice, only probed the rabbit but did not properly imbibe the second blood meal.

The efficiency of PMM-based MALDI-TOF mass spectrometry was further validated by blood meal source determination of field-caught sand fly females, testing its usefulness in actual epidemiological surveillance study. In a blind study on sand flies originating from field collections in Greece, blood meal origin was reliably assigned for almost all (96%) specimens. Moreover, mixed blood meals were identified in two females from this field survey.

Beside blood meal analysis, the actual species identification of engorged females is also very important. As only dissected abdomen is utilized for blood meal origin determination using PMM-based MALDI-TOF mass spectrometry, the rest of the body is still available for sand fly identification and eventually also detection of pathogens like *Leishmania* by other methods. In our study, head and genitalia were slide-mounted for morphological analysis and thorax was used for species determination using MALDI-TOF MS protein profiling which successfully identified all engorged females except one from Greek field survey. Although 12 females had damaged peritrophic matrix, which resulted in visible blood traces in the thorax, 7 were conclusively identified with LSV score higher than 2.0 and four specimens yielded correct species assignment with LSV below 1.8 which was validated by morphology. Only one specimen was not identified at all due to low spectrum quality. These results are in contrast with a recent study, where authors reliably determined only 35% of engorged females and suggested that fresh engorgement of phlebotomine sand flies could compromise successful sand fly species identification by MALDI-TOF MS protein profiling [39]. Our findings however proved that MALDI-TOF MS protein profiling is the proper method not only for species identification of non-engorged specimens, but even of bloodfed sand fly females.

In conclusion, we present a novel approach for blood meal source identification of parasitic arthropods based on peptide mass mapping-based MALDI-TOF mass spectrometry of host-specific hemoglobin peptides. The method was developed on lab-reared specimens and further verified using field-collected samples. Although tested on phlebotomine sand flies, it could be universally applied to other hematophagous insects as we demonstrated by the analysis of experimentally bloodfed *Culex* mosquitoes. The method represents an efficient tool for accurate identification of host blood as it requires a minimal material input and simple and fast sample preparation, especially useful for high numbers of specimens collected during field surveys. Surpassing any currently used DNA- or protein-based methods, our approach allows reliable blood meal source determination until 48h after feeding and moreover, it is capable to detect and correctly identify also mixed blood meals. The specificity of the method enables to distinguish closely related hosts (goat vs. sheep, rodent species) and also to cope with potential hemoglobin isoforms and its allelic variants. Unlike in MALDI-TOF MS protein profiling, a prior creation of database that would include protein profiles of candidate hosts is not needed. The simple, rapid and cost-effective sample preparation that utilizes only abdomen enables to use remaining body parts for simultaneous sand fly species identification by other methods including vouchering of head and genitalia for morphological reference and isolation of DNA for species and blood meal identification by sequencing as well as possible PCR detection of *Leishmania* and other pathogens. All above described advantages of this novel method hopefully make it a promising future tool of choice for blood meal identification in sand flies as well as other hematophagous arthropods.

## Supporting information

S1 Fig MALDI-TOF protein profiles of homogenized female abdomens of *P*. *perniciosus* bloodfed on five different hosts 24 h after feeding. Peaks of host hemoglobin subunit alpha (HBA; about 15 kDa) and hemoglobin subunit beta (HBB; about 16 kDa) dominated in the spectra. For each host the spectra of two females are presented.
(PDF)

S2 Fig MALDI-TOF protein profiles of homogenized female abdomens of *P*. *perniciosus* bloodfed on rabbit at different time points after feeding. The fragments of hemoglobin were produced by blood digestion in the 24-36h PBM time interval. For each time point the spectra of two females are presented.
(PDF)

S3 Fig MALDI-TOF peptide mass maps measured after trypsin digestion of homogenized abdomens of *P*. *perniciosus* fed on grass rat and hamster. The peaks are labelled by corresponding peptide sequences (see Table 1) of host HBA (asterisks) and HBB (circles). The bloodfed females were collected 12 h PBM.
(PDF)

S4 Fig Abdominal peptide mass spectra of *P*. *perniciosus* females at different time points after blood feeding on SKH1 mouse. The peptide signals of mouse hemoglobin are marked by asterisks. The peaks related to digestion of sand fly body become obvious since 48h PBM. For each time point the spectra of two females are presented.
(PDF)

S5 Fig MALDI-TOF protein spectra of homogenized abdomens of *P*. *perniciosus* females fed on SKH1 mouse, rabbit, and on both hosts (mouse and rabbit). The bloodfed females were collected 12 h PBM. HBA: hemoglobin subunit alpha, HBB: hemoglobin subunit beta.
(PDF)

S6 Fig Identification of mixed blood meals. MALDI-TOF mass spectrum of trypsin-digested abdomen of the field-caught specimen EME8 (see Table 3) demonstrating the capability of PMM approach to uncover mixed blood meals. The peptide signals characteristic for each host, which enabled their conclusive identification, are highlighted in grey. The other peaks are common for both the hosts. HBA: hemoglobin subunit alpha, HBB: hemoglobin subunit beta.
(PDF)

S7 Fig Identification of mixed blood meals. Zoomed MALDI-FTICR mass spectrum of trypsin-digested abdomen of the field-caught specimen EME8. The extremely high resolution and mass accuracy of MALDI-FTICR MS analysis allowed distinguishing the overlapping isotopic envelopes of peptides HHGNEFTPVLQADFQK from sheep and HHGSEFTPLLQAEFQK from goat. The error between experimental and theoretical masses for both peptides is below 1 ppm.
(PDF)

S1 Table Comparison of mixed bloodmeal identification at different time points after feeding on SKH1 mouse and rabbit using MALDI-TOF MS protein profiling and PMM-based MALDI-TOF mass spectrometry. Ten females for each time point was subjected to host identification using MALDI-TOF MS protein profiling, five specimens per time point was analyzed using PMM-based MALDI-TOF mass spectrometry.
(PDF)

S2 Table Host identification of field-caught engorged females from Greece using PMM-based MALDI-TOF mass spectrometry. The table includes specimen code, locality, host species, number of detected hemoglobin peptides, and sequence, protonated MW, and MASCOT score of taxa-specific peptides identified by MS/MS sequencing. * Females with the damaged peritrophic matrix.
(PDF)
