# Supplementary figures and images for: A novel MALDI-TOF MS-based method for blood meal identification in insect vectors: A proof of concept study on phlebotomine sand flies
Source: PLoS Negl Trop Dis. 2019 Sep 9;13(9):e0007669. doi: 10.1371/journal.pntd.0007669 (PMC6733444; doi:10.1371/journal.pntd.0007669)

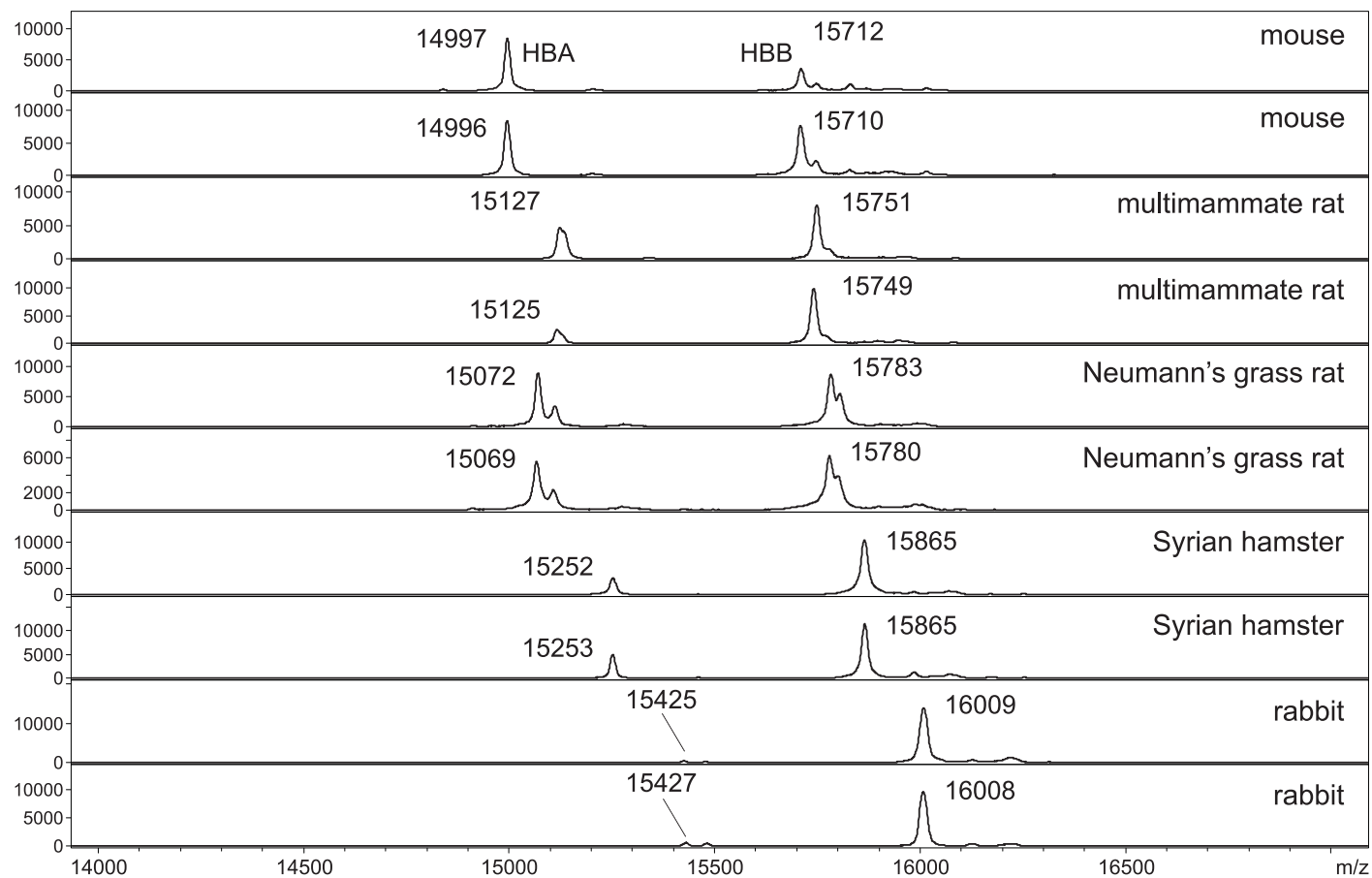

Supplement: S1 Fig — Peaks of host hemoglobin subunit alpha (HBA; about 15 kDa) and hemoglobin subunit beta (HBB; about 16 kDa) dominated in the spectra. For each host the spectra of two females are presented. (PDF) [file pntd.0007669.s001.pdf]

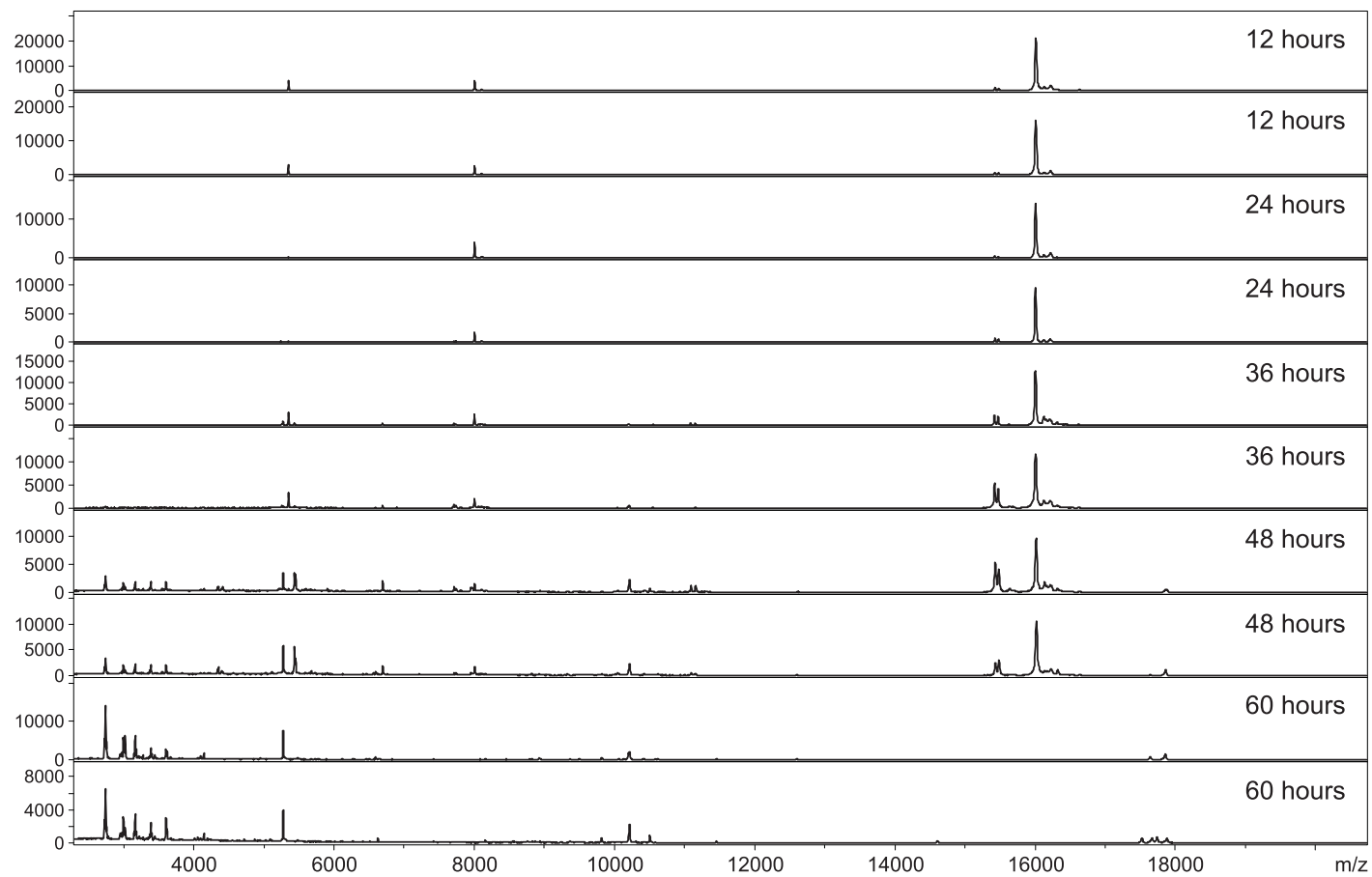

Supplement: S2 Fig — The fragments of hemoglobin were produced by blood digestion in the 24-36h PBM time interval. For each time point the spectra of two females are presented. (PDF) [file pntd.0007669.s002.pdf]

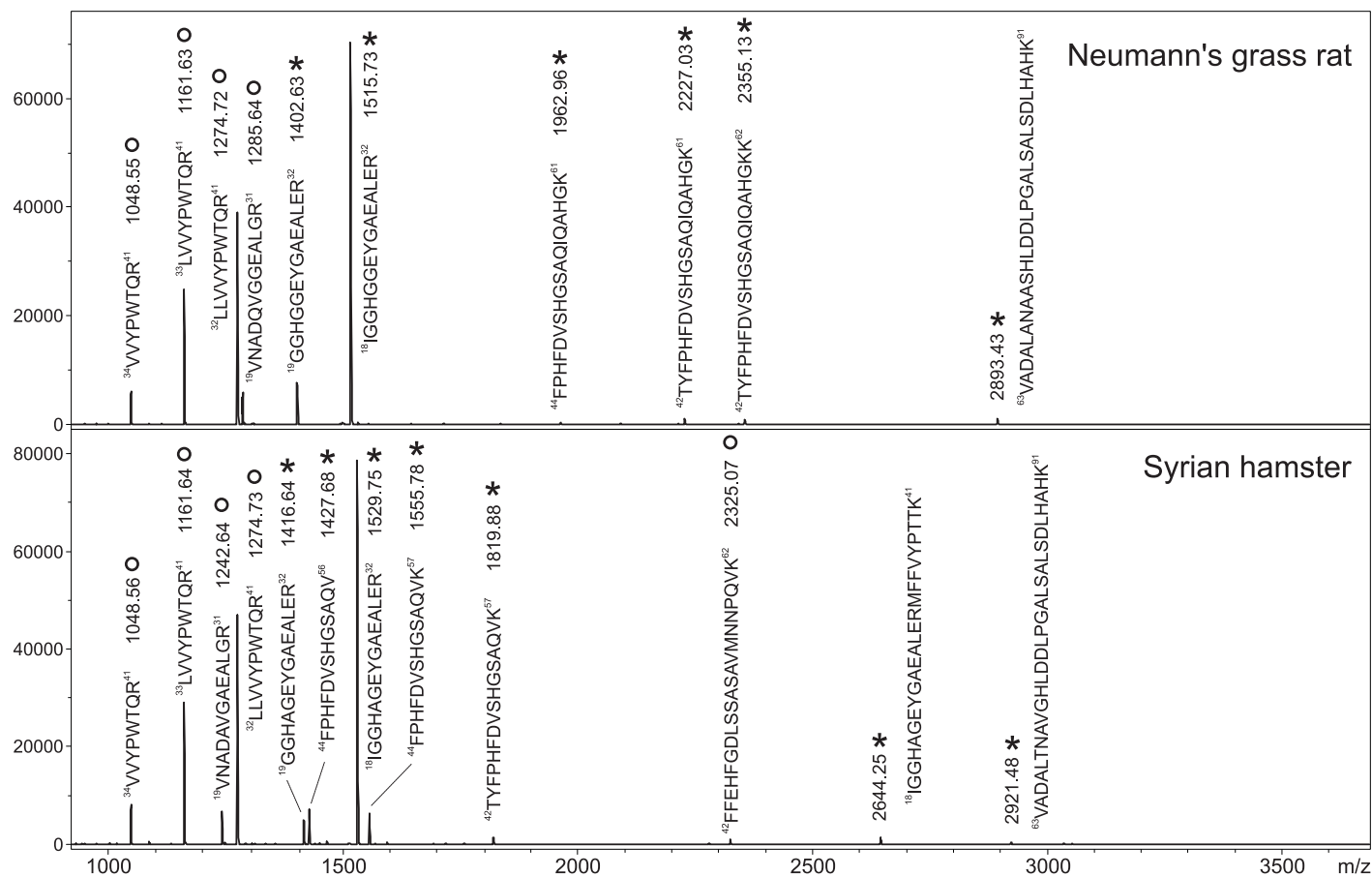

Supplement: S3 Fig — The peaks are labelled by corresponding peptide sequences (see Table 1) of host HBA (asterisks) and HBB (circles). The bloodfed females were collected 12 h PBM. (PDF) [file pntd.0007669.s003.pdf]

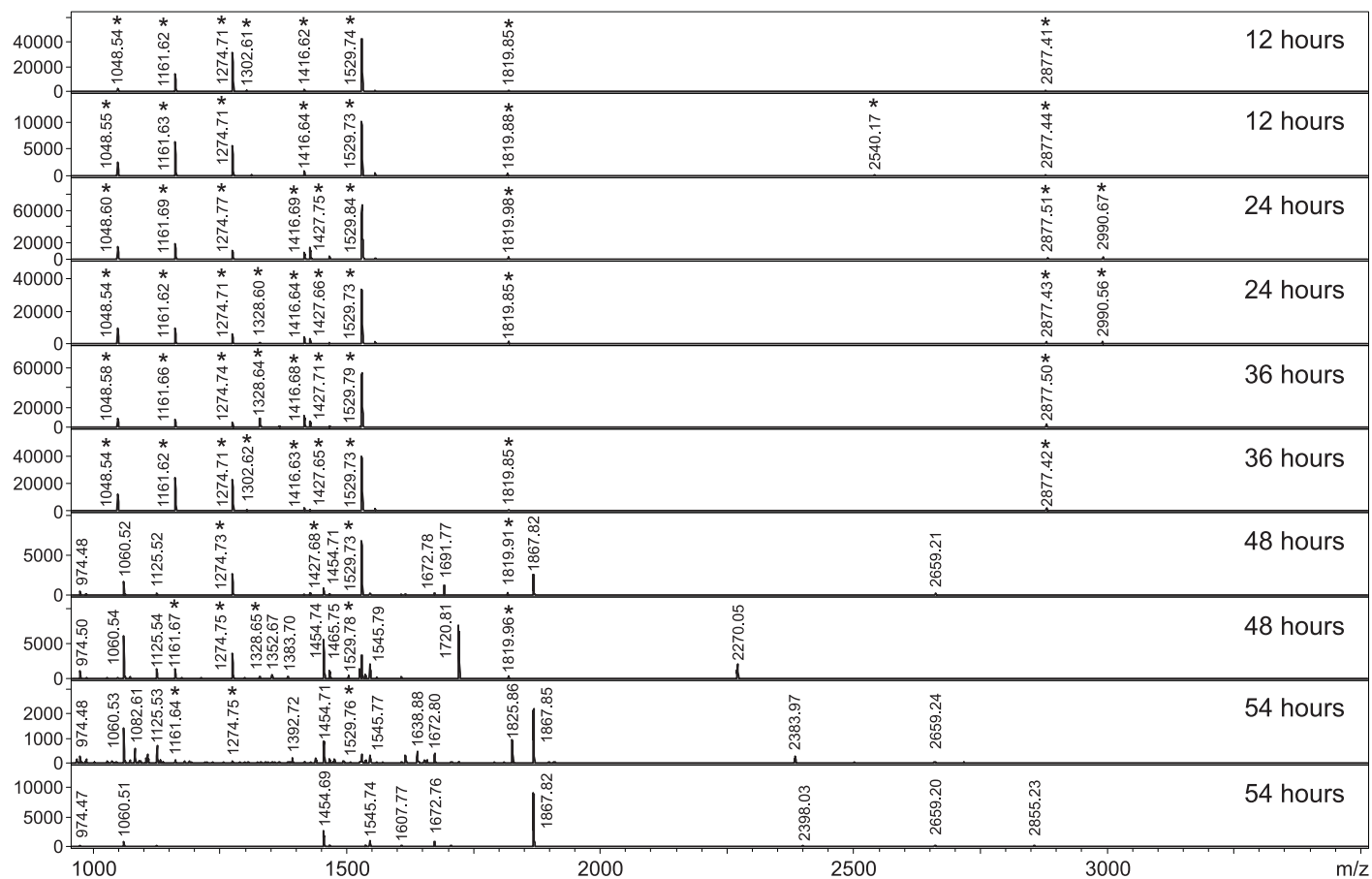

Supplement: S4 Fig — The peptide signals of mouse hemoglobin are marked by asterisks. The peaks related to digestion of sand fly body become obvious since 48h PBM. For each time point the spectra of two females are presented. (PDF) [file pntd.0007669.s004.pdf]

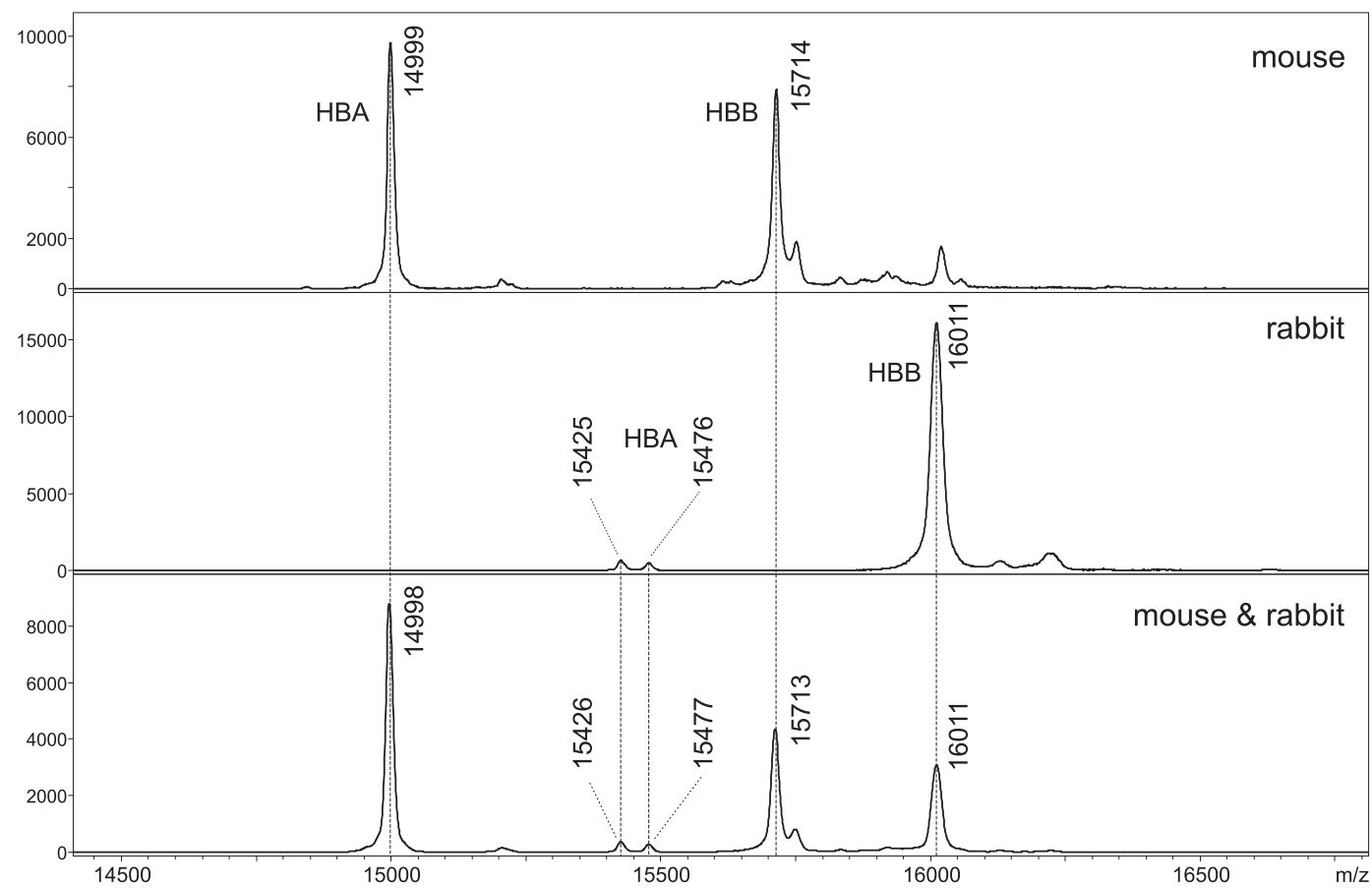

Supplement: S5 Fig — The bloodfed females were collected 12 h PBM. HBA: hemoglobin subunit alpha, HBB: hemoglobin subunit beta. (PDF) [file pntd.0007669.s005.pdf]

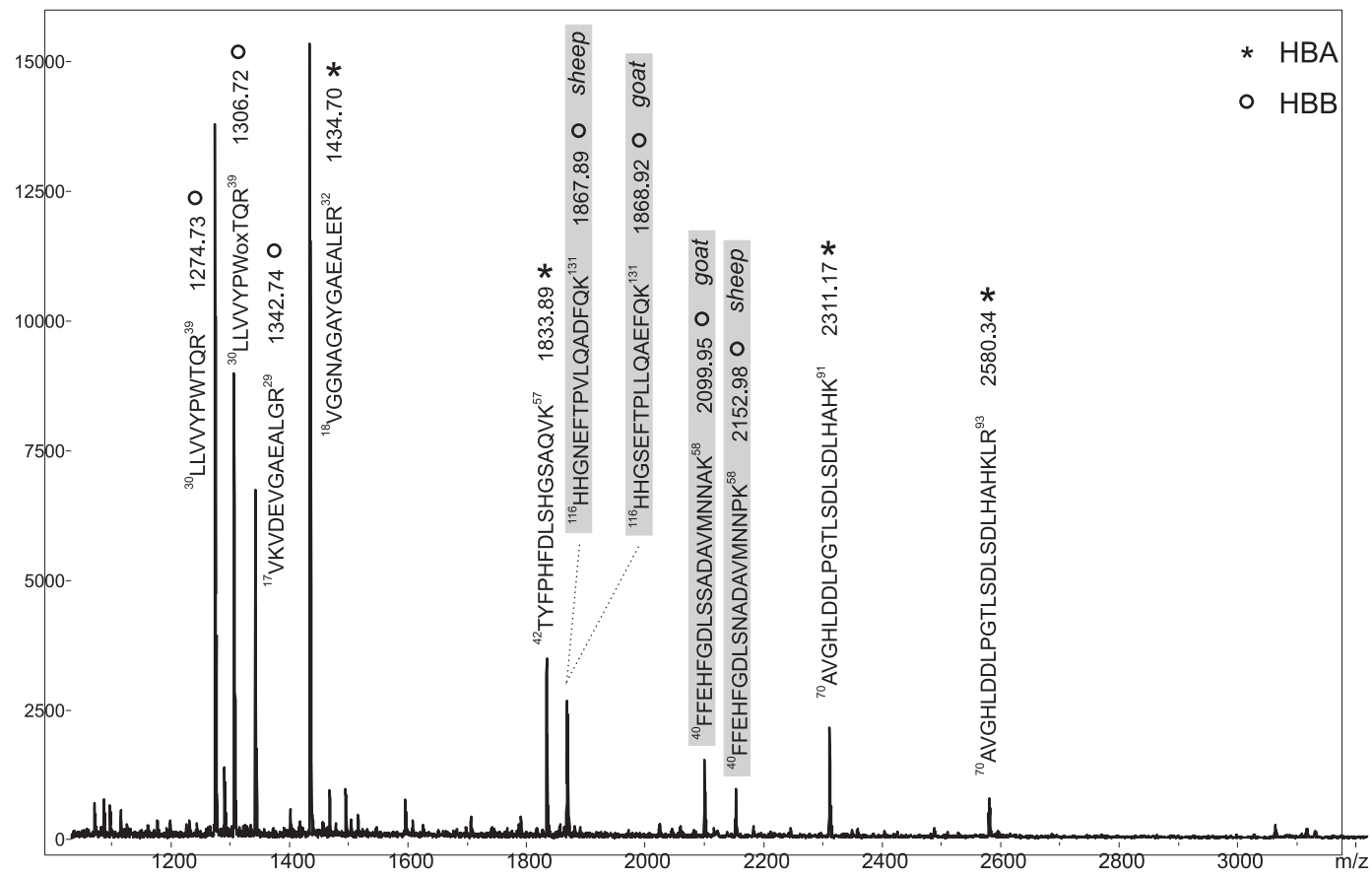

Supplement: S6 Fig — MALDI-TOF mass spectrum of trypsin-digested abdomen of the field-caught specimen EME8 (see Table 3) demonstrating the capability of PMM approach to uncover mixed blood meals. The peptide signals characteristic for each host, which enabled their conclusive identification, are highlighted in grey. The other peaks are common for both the hosts. HBA: hemoglobin subunit alpha, HBB: hemoglobin subunit beta. (PDF) [file pntd.0007669.s006.pdf]

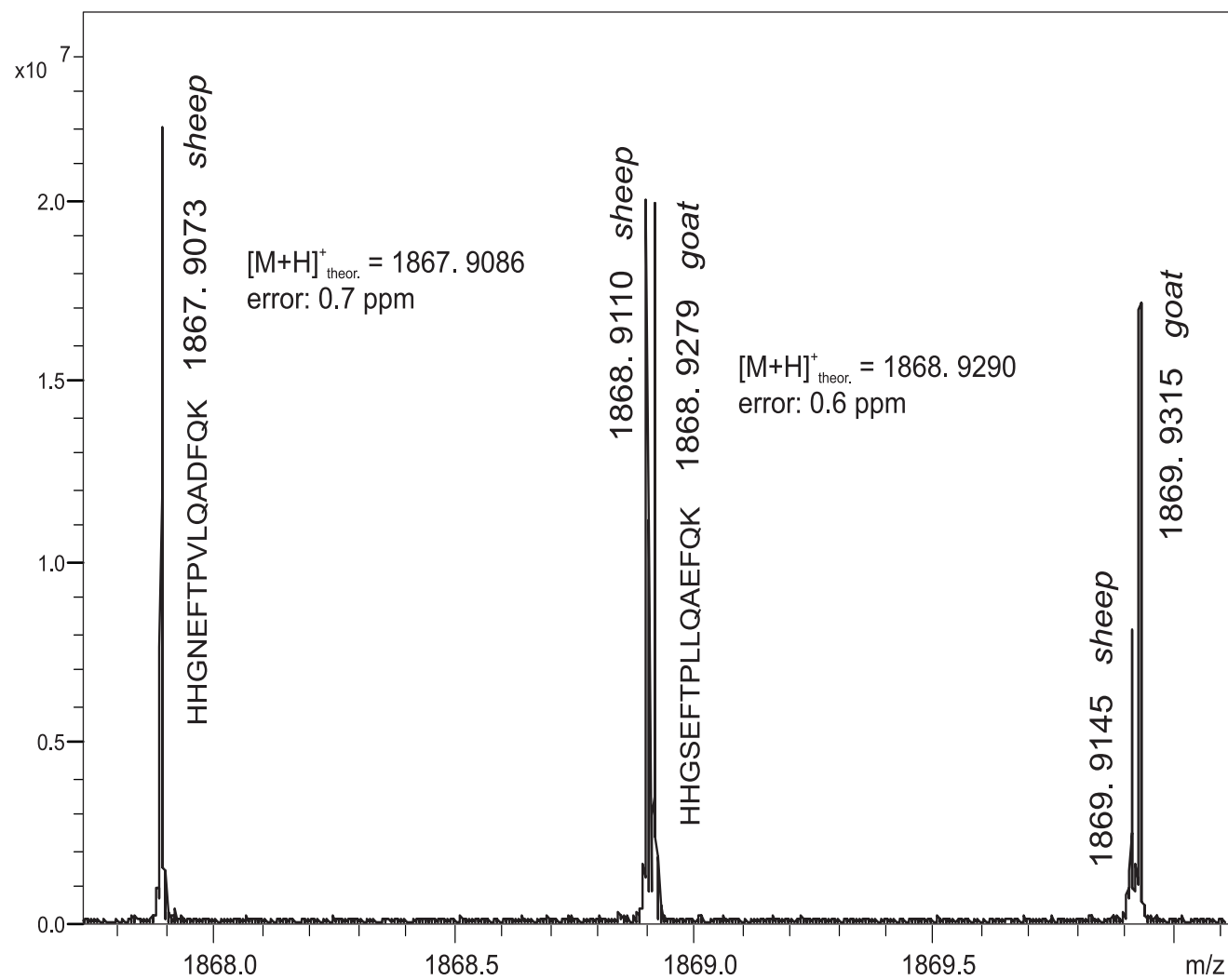

Supplement: S7 Fig — Zoomed MALDI-FTICR mass spectrum of trypsin-digested abdomen of the field-caught specimen EME8. The extremely high resolution and mass accuracy of MALDI-FTICR MS analysis allowed distinguishing the overlapping isotopic envelopes of peptides HHGNEFTPVLQADFQK from sheep and HHGSEFTPLLQAEFQK from goat. The error between experimental and theoretical masses for both peptides is below 1 ppm. (PDF) [file pntd.0007669.s007.pdf]
