# Supplementary material for: A novel MALDI-TOF MS-based method for blood meal identification in insect vectors: A proof of concept study on phlebotomine sand flies
Source: PLoS Negl Trop Dis. 2019 Sep 9;13(9):e0007669. doi: 10.1371/journal.pntd.0007669 (PMC6733444; doi:10.1371/journal.pntd.0007669)
Supplement: S1 Table — Ten females for each time point was subjected to host identification using MALDI-TOF MS protein profiling, five specimens per time point was analyzed using PMM-based MALDI-TOF mass spectrometry. (PDF) [file pntd.0007669.s008.pdf]

	**MALDI-TOF MS protein profiling**			**PMM-based MALDI-TOF MS**		
**PBM time**	both hosts	mouse only	rabbit only	both hosts	mouse only	rabbit only
12 h	0	9	1	3	2	0
24 h	0	3	0	3	2	0
36 h	0	0	0	3	1	1
48 h	0	0	0	0	1	4
54 h	0	0	0	0	0	0
