# Supplementary material for: A novel MALDI-TOF MS-based method for blood meal identification in insect vectors: A proof of concept study on phlebotomine sand flies
Source: PLoS Negl Trop Dis. 2019 Sep 9;13(9):e0007669. doi: 10.1371/journal.pntd.0007669 (PMC6733444; doi:10.1371/journal.pntd.0007669)
Supplement: S2 Table — The table includes specimen code, locality, host species, number of detected hemoglobin peptides, and sequence, protonated MW, and MASCOT score of taxa-specific peptides identified by MS/MS sequencing. * Females with the damaged peritrophic matrix. (PDF) [file pntd.0007669.s009.pdf]

Code	Locality	PMM host identification	No. of HB peptides	Peptides identified by MS/MS sequencing	MH^+ [Da]	Mascot score
EAG1	Agriacona	*Capra hircus*	10	HHGSEFTPLLQAEFQK	1868.9	86
				FFEHFGDLSSADAVMNNAK	2099.9	105
				AVGHLDDLPGTLSDLSDLHAHKLR	2580.3	102
EAG21*	Agriacona	*Capra hircus*	13	VGGNAGAYGAEALER	1434.7	97
				HHGSEFTPLLQAEFQK	1868.9	102
				FFEHFGDLSSADAVMNNAK	2099.9	133
EAG22	Agriacona	*Capra hircus*	11	HHGSEFTPLLQAEFQK	1868.9	61
				FFEHFGDLSSADAVMNNAK	2099.9	122
				AVGHLDDLPGTLSDLSDLHAHK	2311.2	117
EAG23	Agriacona	*Canis lupus familiaris*	11	VNVDEVGGEALGR	1314.7	59
				IGGHAGDYGGEALDR	1487.7	124
				TYFPHFDLSPGSAQVK	1793.9	82
				FFDSFGDLSTPDAVMSNAK	2048.9	129
ELE6	Levidi	*Ovis aries*	12	VGGNAGAYGAEALER	1434.7	121
				TYFPHFDLSHGSAQVK	1833.9	91
				FFEHFGDLSNADAVMNNPK	2153.0	127
				AVGHLDDLPGTLSDLSDLHAHK	2311.2	108
				AVGHLDDLPGTLSDLSDLHAHKLR	2580.4	100
ELE7	Levidi	*Canis lupus familiaris*	9	VNVDEVGGEALGR	1314.7	51
				IGGHAGDYGGEALDR	1487.7	85
				TYFPHFDLSPGSAQVK	1793.9	43
				FFDSFGDLSTPDAVMSNAK	2048.9	119
ELE8	Levidi	*Ovis aries*	12	HHGNEFTPVLQADFQK	1867.9	91
				FFEHFGDLSNADAVMNNPK	2153.0	126
ELE9	Levidi	*Ovis aries*	13	HHGNEFTPVLQADFQK	1867.9	87
				FFEHFGDLSNADAVMNNPK	2153.0	106
ELE10	Levidi	*Ovis aries*	13	HHGNEFTPVLQADFQK	1867.9	79
				FFEHFGDLSNADAVMNNPK	2153.0	109
ELE11*	Levidi	*Ovis aries*	14	HHGNEFTPVLQADFQK	1867.9	93
				FFEHFGDLSNADAVMNNPK	2153.0	113
ELO1	Leontari	*Ovis aries*	10	VKVDEVGAEALGR	1342.7	83
				VGGNAGAYGAEALER	1434.7	123
				HHGNEFTPVLQADFQK	1867.9	93
				FFEHFGDLSNADAVMNNPK	2153.0	143
				AVGHLDDLPGTLSDLSDLHAHKLR	2580.4	73
ELV31*	Levidi / Vlacherna	n.d.				
ELV32*	Levidi / Vlacherna	*Capra hircus*	10	HHGSEFTPLLQAEFQK	1868.9	90
				FFEHFGDLSSADAVMNNAK	2099.9	133
EME1	Metamorphose	*Ovis aries*	9	VKVDEVGAEALGR	1342.7	59
				VGGNAGAYGAEALER	1434.7	77
				TYFPHFDLSHGSAQVK	1833.9	42
				HHGNEFTPVLQADFQK	1867.9	65
EME2	Metamorphose	*Ovis aries*	12	VGGNAGAYGAEALER	1434.7	118
				HHGNEFTPVLQADFQK	1867.9	101
				FFEHFGDLSNADAVMNNPK	2153.0	94
				AVGHLDDLPGTLSDLSDLHAHKLR	2580.4	45
EME3	Metamorphose	*Ovis aries*	12	VGGNAGAYGAEALER	1434.7	117
				HHGNEFTPVLQADFQK	1867.9	117
				FFEHFGDLSNADAVMNNPK	2153.0	127
				AVGHLDDLPGTLSDLSDLHAHK	2311.2	154
EME4	Metamorphose	*Ovis aries*	11	VKVDEVGAEALGR	1342.7	77
				VGGNAGAYGAEALER	1434.7	82
				HHGNEFTPVLQADFQK	1867.9	85
				FFEHFGDLSNADAVMNNPK	2153.0	134
EME5	Metamorphose	*Ovis aries*	12	VGGNAGAYGAEALER	1434.7	123
				HHGNEFTPVLQADFQK	1867.9	110

				FFEHFGDLSNADAVMNNPK	2153.0	121
				AVGHLDDLPGTLSDLSDLHAHKLR	2580.4	47
EME6	Metamorphose	*Ovis aries*	12	VGGNAGAYGAEALER	1434.7	123
				HHGNEFTPVLQADFQK	1867.9	95
				FFEHFGDLSNADAVMNNPK	2153.0	124
				AVGHLDDLPGTLSDLSDLHAHK	2311.2	154
EME7	Metamorphose	*Ovis aries*	10	HHGNEFTPVLQADFQK	1867.9	89
				FFEHFGDLSNADAVMNNPK	2153.0	66
EME8	Metamorphose	*Ovis aries*	12	HHGNEFTPVLQADFQK	1867.9	59
		+		FFEHFGDLSNADAVMNNPK	2153.0	126
		Capra hircus		HHGSEFTPLLQAEFQK	1868.9	73
				FFEHFGDLSSADAVMNNAK	2099.9	143
EME9	Metamorphose	*Ovis aries*	9	VGGNAGAYGAEALER	1434.7	66
				HHGNEFTPVLQADFQK	1867.9	66
EME10	Metamorphose	*Ovis aries*	11	HHGNEFTPVLQADFQK	1867.9	94
				FFEHFGDLSNADAVMNNPK	2153.0	112
EME11	Metamorphose	*Ovis aries*	11	HHGNEFTPVLQADFQK	1867.9	117
				FFEHFGDLSNADAVMNNPK	2153.0	119
EME12	Metamorphose	*Ovis aries*	11	HHGNEFTPVLQADFQK	1867.9	99
				FFEHFGDLSNADAVMNNPK	2153.0	121
EME13*	Metamorphose	*Capra hircus*	9	HHGSEFTPLLQAEFQK	1868.9	86
				FFEHFGDLSSADAVMNNAK	2099.9	65
EME14	Metamorphose	*Ovis aries*	9	HHGNEFTPVLQADFQK	1867.9	80
				FFEHFGDLSNADAVMNNPK	2153.0	41
EME15	Metamorphose	*Ovis aries*	9	VGGNAGAYGAEALER	1434.7	116
				HHGNEFTPVLQADFQK	1867.9	56
EME16	Metamorphose	*Ovis aries*	11	HHGNEFTPVLQADFQK	1867.9	106
				FFEHFGDLSNADAVMNNPK	2153.0	126
EME17	Metamorphose	*Ovis aries*	12	HHGNEFTPVLQADFQK	1867.9	100
				FFEHFGDLSNADAVMNNPK	2153.0	129
EME18	Metamorphose	*Ovis aries*	11	HHGNEFTPVLQADFQK	1867.9	113
				FFEHFGDLSNADAVMNNPK	2153.0	110
EME19	Metamorphose	*Ovis aries*	12	HHGNEFTPVLQADFQK	1867.9	104
				FFEHFGDLSNADAVMNNPK	2153.0	118
EME20*	Metamorphose	*Ovis aries*	11	HHGNEFTPVLQADFQK	1867.9	52
				FFEHFGDLSNADAVMNNPK	2153.0	107
				AVGHLDDLPGTLSDLSDLHAHKLR	2580.4	73
EPA10	Palaio Pyrgos	*Capra hircus*	12	HHGSEFTPLLQAEFQK	1868.9	68
				FFEHFGDLSSADAVMNNAK	2099.9	131
EPE1*	Pelopio	*Ovis aries*	11	VKVDEVGAEALGR	1342.7	79
				VGGNAGAYGAEALER	1434.7	123
				HHGNEFTPVLQADFQK	1867.9	103
				FFEHFGDLSNADAVMNNPK	2153.0	131
EPE2	Pelopio	*Ovis aries*	11	VGGNAGAYGAEALER	1434.7	120
				HHGNEFTPVLQADFQK	1867.9	80
				FFEHFGDLSNADAVMNNPK	2153.0	88
				AVGHLDDLPGTLSDLSDLHAHKLR	2580.4	99
EPK1	Pakia	*Ovis aries*	12	VKVDEVGAEALGR	1342.7	84
				VGGNAGAYGAEALER	1434.7	125
				HHGNEFTPVLQADFQK	1867.9	68
				FFEHFGDLSNADAVMNNPK	2153.0	105
EPK2	Pakia	*Ovis aries*	11	VGGNAGAYGAEALER	1434.7	115
				HHGNEFTPVLQADFQK	1867.9	93
				FFEHFGDLSNADAVMNNPK	2153.0	135
EPK3*	Pakia	n.d.				
EPK4	Pakia	*Ovis aries*	10	HHGNEFTPVLQADFQK	1867.9	80
				FFEHFGDLSNADAVMNNPK	2153.0	105
EPK5	Pakia	*Canis lupus familiaris*	8	VNVDEVGGEALGR	1314.7	43
				IGGHAGDYGGEALDR	1487.7	89
				FFDSFGDLSTPDAVMSNAK	2048.9	76
EPK6	Pakia	*Ovis aries*	12	HHGNEFTPVLQADFQK	1867.9	82
		+		FFEHFGDLSNADAVMNNPK	2153.0	117
		Capra hircus		HHGSEFTPLLQAEFQK	1868.9	40

				FFEHFGDLSSADAVMNNAK	2099.9	132
EPO1	Agriacona	*Gallus gallus*	9	AASHQEEFGAEALTR<br>IAGHAEEYGAETLER<br>TYFPHFDLSHGSAQIK<br>TYFPHFDLSPGSDQVR<br>NVDNLSQAMAELSNLHAYNLR	1616.8<br>1645.8<br>1847.9<br>1865.9<br>2373.3	119<br>108<br>52<br>79<br>118
EPO14	Agriacona	*Ovis aries*	9	HHGNEFTPVLQADFQK<br>FFEHFGDLSNADAVMNNPK	1867.9<br>2153.0	70<br>64
EPP1	Papari	*Ovis aries*	12	HHGNEFTPVLQADFQK<br>FFEHFGDLSNADAVMNNPK	1867.9<br>2153.0	103<br>126
EPP2	Papari	*Ovis aries*	11	HHGNEFTPVLQADFQK<br>FFEHFGDLSNADAVMNNPK	1867.9<br>2153.0	111<br>134
EPP3	Papari	*Ovis aries*	10	HHGNEFTPVLQADFQK<br>FFEHFGDLSNADAVMNNPK	1867.9<br>2153.0	87<br>107
EPP4	Papari	*Ovis aries*	10	HHGNEFTPVLQADFQK<br>FFEHFGDLSNADAVMNNPK	1867.9<br>2153.0	120<br>128
EPP5*	Papari	*Ovis aries*	11	HHGNEFTPVLQADFQK<br>FFEHFGDLSNADAVMNNPK	1867.9<br>2153.0	113<br>120
EPT1*	Asteri	*Ovis aries*	10	VKVDEVGAEALGR<br>VGGNAGAYGAEALER<br>HHGNEFTPVLQADFQK<br>FFEHFGDLSNADAVMNNPK	1342.7<br>1434.7<br>1867.9<br>2153.0	39<br>89<br>71<br>110
EPT2	Asteri	*Ovis aries*	12	VGGNAGAYGAEALER<br>HHGNEFTPVLQADFQK<br>FFEHFGDLSNADAVMNNPK<br>AVGHLDDLPGTLSDLSDLHAHKLR	1434.7<br>1867.9<br>2153.0<br>2580.4	104<br>114<br>133<br>121
EPT20*	Asteri	*Ovis aries*	11	HHGNEFTPVLQADFQK<br>FFEHFGDLSNADAVMNNPK	1867.9<br>2153.0	95<br>119
EPT21	Asteri	*Ovis aries*	12	HHGNEFTPVLQADFQK<br>FFEHFGDLSNADAVMNNPK	1867.9<br>2153.0	115<br>136
EST1*	Stefania	*Capra hircus*	9	VGGNAGAYGAEALER<br>HHGSEFTPLLQAEFQK<br>FFEHFGDLSSADAVMNNAK	1434.7<br>1868.9<br>2099.9	44<br>55<br>49

* Females with the damaged peritrophic matrix.
